# Addiction Susceptibility: Genetic Factors, Personality Traits, and Epigenetic Interactions with the Gut Microbiome

**DOI:** 10.3390/genes16121447

**Published:** 2025-12-03

**Authors:** Alejandro Borrego-Ruiz, Juan J. Borrego

**Affiliations:** 1 Departamento de Psicología Social y de las Organizaciones, Universidad Nacional de Educación a Distancia (UNED), 28040 Madrid, Spain; a.borrego@psi.uned.es; 2 Departamento de Microbiología, Universidad de Málaga, 29071 Málaga, Spain

**Keywords:** addiction, addictive behavior, substance use disorders, susceptibility, vulnerability, genes, personality, epigenetics, gut microbiome

## Abstract

Despite valuable insights into the individual roles of genetic factors and personality traits, their combined contribution to addiction susceptibility remains insufficiently characterized. Within this framework, the potential influence of epigenetic mechanisms, particularly those mediated by the gut microbiome, also remains underexplored. This comprehensive review aims to address these gaps in an integrative manner by examining: (i) the association of gene regulation with personality traits; (ii) the genetics of substance use disorders; (iii) the roles of genes and personality in addiction; and (iv) epigenetic influences on addiction, with a particular focus on the role of the gut microbiome. Genetic influences on personality act primarily via regulatory variants that modulate gene expression during neurodevelopment, shaping cognitive, emotional, and behavioral traits that contribute to individual differences. Substance use disorders share partially overlapping genetic foundations, with specific loci, heritability estimates, and causal pathways differing across substances, reflecting both shared vulnerability and substance-specific genetic influences on addiction susceptibility. Impulsivity, novelty-seeking, and stress responsiveness are heritable personality traits that interact to shape susceptibility to substance use disorders, with genetic factors modulating risk across different forms of addiction. Environmental factors, early-life stress, and social influences interact with the gut microbiome to shape neurobiological and behavioral pathways that modulate addiction risk. These interactions highlight the multifactorial nature of substance use disorders, in which epigenetic, microbial, and psychosocial mechanisms converge to influence susceptibility, progression, and maintenance of addictive behaviors.

## 1. Introduction

Addiction is widely recognized as a complex behavioral disorder that reflects an interplay between genetic vulnerability and environmental influences. Depending on the object or element to which an individual is addicted, it is possible to differentiate between substance addiction or behavioral addiction, with the latter involving compulsive engagement in specific behaviors (e.g., gambling, internet use, sexual activity), rather than the consumption of a psychoactive substance, and showing similar psychological consequences and personality traits (e.g., higher impulsivity), but generally lacking physical withdrawal symptoms [1,2]. According to the DSM-5, substance use disorders (SUDs) are diagnosed when individuals meet at least two out of eleven possible criteria within a 12-month period. The severity of the disorder is determined by the number of criteria endorsed: two to three indicating mild, four to five indicating moderate, and six or more reflecting severe SUD [3]. These diagnostic indicators capture a range of substance-related difficulties, including escalating patterns of use, repeated failed attempts to reduce or discontinue use, persistent craving, tolerance, withdrawal, and continued consumption despite adverse physical, psychological, or social consequences. In contrast, the earlier DSM-IV framework separated substance abuse from substance dependence. Under that system, a diagnosis of abuse required at least one of four abuse-related symptoms, whereas dependence was defined by the presence of at least three of seven dependence criteria [4]. Interestingly, evidence from Compton et al. [5] suggested that a DSM-5 threshold of four or more criteria, which corresponds to a moderate-level SUD, aligned most closely with the DSM-IV diagnosis of dependence for alcohol, cocaine, and opioid use disorders. From a neurobiological perspective, the mesolimbic dopamine system provides one of the most comprehensive explanatory frameworks for addictive processes. This circuit, originating in the ventral tegmental area (VTA) and projecting to regions such as the nucleus accumbens (NAc) and orbitofrontal cortex (OFC), mediates the motivational and reinforcing effects of addictive agents [6]. By exploiting the role of dopamine in reward prediction and action learning, addictive substances and behaviors establish powerful associations between substance intake and subjective pleasure. Once formed, these associations can be reactivated by conditioned cues, perpetuating compulsive substance-seeking and relapse despite awareness of harmful consequences. Nevertheless, addiction involves a complex neurochemical and physiological interplay. Other neurotransmitters, including serotonin, endogenous opioids (e.g., endorphins, enkephalins), acetylcholine, γ-aminobutyric acid (GABA), and glutamate, participate in different phases and processes of addictive behavior [7]. In addition, individual predisposition to addiction can be promoted by stressful environmental conditions, such as social isolation and deprivation of parental care, which modulate neurochemical systems and heighten vulnerability to compulsive use [6]. From an evolutionary perspective, these mechanisms may operate within a progressively developed brain structure in which monoaminergic and cholinergic systems underlie cognitive and motivational functions [6].

### 1.1. Etiology and Risk Factors

Current evidence emphasizes that addiction does not arise merely from repeated exposure to a substance or behavior, but rather from the convergence of intrinsic factors (e.g., sex, age, genotype), extrinsic influences (e.g., education, availability of substances), and properties of the addictive stimulus itself (e.g., route of administration, pharmacokinetics, and psychoactive characteristics) [7]. Among the main etiological contributors of addictive behavior, genetic influences are particularly relevant, with multiple genes implicated in modulating susceptibility [8]. In addition to genetic determinants, individual susceptibility is shaped by several domains: (i) neurobiological factors, such as heightened dopaminergic activity in the striatum combined with diminished prefrontal regulation of subcortical motivational and affective responses [8]; (ii) psychiatric contributors, including comorbid mental disorders such as depression and anxiety [9,10]; (iii) psychological vulnerabilities, with chronic stress, impulsivity, and low self-esteem linked to addiction risk [11,12]; (iv) environmental and social determinants, such as unsupportive relationships, limited social interaction, adverse childhood experiences (ACEs), and early exposure to substances [13,14,15,16,17]; and (v) motivational dynamics, in which intrinsic motives (e.g., curiosity, novelty-seeking) and extrinsic motives (e.g., reinforcement from substance use or avoidance of withdrawal symptoms) influence both initiation and maintenance of addictive behaviors [18]. Within this multifactorial framework, personality may constitute a key dimension, as their association with specific brain systems and genetic determinants has been studied to elucidate pathways of vulnerability and resilience to SUDs [19].

### 1.2. Personality Traits

Personality traits are generally understood as patterns of cognition, emotion, and behavior that remain consistent across diverse situations and over time. Different theoretical frameworks diverge on the exact number or structure of traits required to capture individual differences, but they converge on the view that personality can be reduced to a limited set of fundamental dimensions with a heritable basis [20]. Among the various models proposed, the Big Five taxonomy has gained prominence, as it organizes personality into five broad domains. These dimensions, which are observable from early developmental stages, relatively stable throughout the lifespan, and associated with biological processes, constitute predictors of behavior and of long-term outcomes [21]. Although terminology can vary, the dimensions are most frequently identified as neuroticism (versus emotional stability), extraversion (versus introversion), openness to experience, agreeableness, and conscientiousness [22]. However, the notion of an “addictive personality” does not have empirical support within psychiatric nosology. Specifically, it is absent from the DSM-5 and does not qualify as a diagnostic entity [3]. In fact, the term has been criticized for its lack of precision, as well as for the potential to foster misunderstanding and undermine the effectiveness of treatment strategies for individuals with SUDs [23]. Nevertheless, it could be considered plausible to use the expression as a heuristic to describe clusters of psychological traits that may increase vulnerability to addiction. Personality characteristics relevant to addiction appear to differ between males and females, which could be attributed to the influence of biological and psychosocial factors. Neuroanatomical studies, for instance, have revealed sex-based differences in brain regions implicated in emotion and motivation: females generally show larger hippocampal volumes, linked to estrogen synthesis, while males display larger amygdalae, where androgen concentrations are highest [24]. These neurobiological distinctions are reflected in psychological profiles. Women typically score higher on traits associated with anxiety and affect regulation, such as neuroticism and harm avoidance [25,26]. Men, by contrast, more often exhibit higher levels of persistence, openness to experience, self-confidence, and self-esteem [27,28]. Furthermore, women have been reported to show greater conscientiousness, reward dependence, self-transcendence, and cooperativeness, but lower self-directedness when compared to men [25,29].

### 1.3. Genetics and Epigenetics

Despite evidence indicating that SUDs have a heritable component, efforts to identify specific alleles that reliably predict addiction vulnerability have produced limited success [30,31]. This difficulty likely reflects the multifactorial nature of addiction, in which genetic influences may exert greater importance under certain environmental conditions or within particular subgroups of the population [32]. Genetic variation can shape the density and distribution of neural receptors, thereby modulating the sensitivity of individuals to psychoactive substances [33]. Moreover, polymorphisms in hepatic enzymes alter drug metabolism rates, which in turn affect both substance response and pharmacological treatment efficacy [33]. These insights have given rise to the field of pharmacogenetics, which seeks to optimize therapeutic interventions by customizing medications to the genetic profile of a specific subject [34]. In addition to genetic variation, epigenetic regulation has been increasingly recognized as a pivotal determinant of addiction risk. Epigenetics involves heritable modifications in gene expression that arise without changes to the underlying DNA sequence. Key mechanisms include DNA methylation, post-translational histone modifications, and regulation by non-coding RNAs. These epigenetic processes can be influenced by exposure to psychoactive substances, leading to persistent alterations in neural function, stress reactivity, and behavior [34].

Adolescence represents a critical developmental period during which environmental exposures can substantially shape whether inherited vulnerabilities manifest in addictive behaviors [14,35]. Indeed, during adolescence values and judgment are not yet fully developed, and curiosity and the inclination toward novel experiences are especially pronounced. This is of particular concern because earlier initiation of substance use may increase the likelihood that such use will progress to established dependence [18]. Thus, the interaction between inherited predisposition and environmental context, commonly referred to as gene–environment interaction, illustrates how genotypic differences can modify the influence of environmental exposures on behavioral outcomes [36].

### 1.4. The Gut–Brain Axis

Growing evidence indicates that SUDs are closely linked to alterations in the gut microbiome (GM) [37,38]. Substance-induced changes in microbial composition (i.e., dysbiosis) can interact with the genetic vulnerability of individuals, establishing a feedback loop that promotes both the initiation and persistence of addictive behaviors [39]. Central to this connection is the gut–brain axis (GBA), consisting of a bidirectional communication system through which gut microorganisms, their metabolites, and intestinal mucosal interactions influence neural activity and behavioral outcomes. In particular, recent findings highlight the contribution of this pathway to opioid use disorder (OUD) [40]. Disruptions in the GM have also been associated with modifications in the expression of striatal dopamine receptors, which appear to correlate with compulsive alcohol-seeking behaviors in animal models [41,42]. Additional preclinical research provides compelling support for this relationship. For instance, the transplantation of enteric microorganisms from alcohol-exposed mice into healthy controls not only altered microbial community structure but also induced behavioral markers consistent with alcohol withdrawal–related anxiety [43]. Although the mechanisms through which gut microorganisms shape responses to drugs remain incompletely understood, microbial products, including short-chain fatty acids (SCFAs), tryptophan metabolites, bile acids (BAs), and neurotransmitters, are thought to contribute by modulating blood–brain barrier (BBB) permeability, immune activation, neuronal signaling, and gene expression [37].

### 1.5. Genomic Technologies

At present, a wide range of genomic technologies and large-scale datasets are available that allow researchers to better connect molecular processes with human phenotypes [44]. For social scientists, these resources provide both opportunities and methodological challenges. Collaborations with molecular genomic researchers open the possibility of combining traditional psychometric and social science measures with genomic information in a meaningful way. In this respect, several categories of genomic data relevant to the study of gene regulation are particularly valuable, as they make it possible to: (i) prioritize disease-associated non-coding variants that may play a causal role in the genetic basis of complex traits or function as useful biomarkers, and (ii) identify genes and biological pathways involved in development, pathology, and environmental responses [45,46,47,48]. Among the main functional genomics approaches there is a variety to take into consideration. (i) RNA sequencing (RNA-seq), which is a next-generation sequencing (NGS) tool that enables the characterization and quantification of the full spectrum of RNA molecules in a biological sample, including at the single-cell level. By analyzing the transcriptome, which constitutes the complete set of transcripts, from mRNA and rRNA to tRNA and non-coding RNAs, researchers obtain a dynamic snapshot of gene expression and regulation at a specific moment [49]. (ii) DNase I–seq [50] and ATAC-seq [51], which identify accessible chromatin regions through DNase I digestion or transposase insertion, followed by sequencing. (iii) Chromatin immunoprecipitation followed by sequencing (ChIP-seq), which allows the mapping of genomic sites bound by regulatory proteins [52], as well as histone modifications associated with either active or repressed chromatin states [53]. Importantly, these epigenetic signatures can shift in response to environmental factors [54]. (iv) Three-dimensional genomics approaches, which permit the association of non-coding single-nucleotide polymorphisms (SNPs) with candidate target genes by identifying chromatin interactions [55]. (v) Massively parallel reporter assays (MPRAs), which leverage DNA sequencing to evaluate the regulatory activity of thousands of DNA sequences simultaneously, a strategy often used to identify non-coding variants that alter regulatory capacity [56]. Collectively, these functional genomics approaches offer unprecedented understanding of the molecular regulation of human traits and behaviors, providing a foundation to investigate how these mechanisms may intersect with personality and addiction-related patterns.

### 1.6. Aim of the Review

Within the current global landscape, addiction constitutes a pressing societal challenge and an issue of undeniable complexity, as exemplified by the devastating impact of the recent fentanyl crisis, which has been amplified by its covert adulteration of other substances and the consequent unawareness of users regarding its extreme potency [57]. Consequently, the progressive elucidation of its underlying determinants is pivotal for guiding effective interventions and public health responses. Despite valuable insights into the individual roles of genetic factors and personality traits, their combined contribution to addiction susceptibility remains insufficiently characterized. Within this framework, the potential influence of epigenetic mechanisms, particularly those mediated by the GM, also remains underexplored. This comprehensive review aims to address these gaps in an integrative manner by examining (i) the association of gene regulation with personality traits; (ii) the genetics of substance use disorders; (iii) the roles of genes and personality in addiction; and (iv) epigenetic influences on addiction, with a particular focus on the role of the GM.

## 2. Association of Gene Regulation with Personality Traits

Genes influence personality and temperament [58]. Variations in specific genes have been associated with traits such as extraversion, neuroticism, adaptability, and other psychological characteristics that shape cognition and behavior. Thus, personality is largely affected by genetic factors that regulate and integrate dynamic functions essential for responding to environmental circumstances [59,60]. These include mechanisms underlying energy balance, neural development, neurogenesis, neurotransmission, neuroprotection, synaptic plasticity, stress regulation, resilience, and overall brain health across the lifespan. Although genes relevant to personality are widely expressed in the brain, genetic diversity modulates key biological pathways, particularly those related to cellular energy production, circadian regulation, and regenerative capacity [61,62].

Although accumulating evidence suggests that personality and other individual difference traits have a genetic basis, only a limited number of specific variants have been consistently linked to these characteristics to date. This scarcity of findings is generally attributed to the highly polygenic nature of such traits [63]. According to the evolutionary neutral theory, genetic variants with substantial effects are expected to be uncommon, whereas the majority of phenotypic diversity arises from numerous common variants each exerting small influences [64]. Early research into the genetic underpinnings of personality primarily focused on candidate genes. However, with the expansion of computational resources, the field has shifted toward approaches that consider the genome at large rather than targeting predefined loci. In contrast to candidate gene studies, which were constrained by prior biological assumptions, genome-wide association studies (GWASs) adopt a hypothesis-free framework, enabling the detection of links across the entire genome.

GWAS have become an essential tool in behavioral genetics, offering valuable insights into the molecular foundations of psychological traits [45]. One of the most significant discoveries from GWAS is that much of the genetic variation linked to behavioral and cognitive traits lies outside protein-coding regions of the genome [65]. These non-coding regions are thought to encompass hundreds of thousands of regulatory elements that control gene activity [66]. Such findings suggest that the genetic underpinnings of complex human traits are largely influenced by variants that affect transcriptional regulation, rather than through direct modifications to protein-coding sequences [45]. Variants that influence gene expression have been implicated in diverse behavioral phenotypes and psychiatric conditions, including bipolar disorder (BD), schizophrenia, autism spectrum disorder (ASD), and personality-related traits such as neuroticism [48,67,68,69].

SNP refers to a variation at a single-nucleotide site within the genome. Evidence indicates that SNPs are associated with cognitive functioning as well as vulnerability to mental health conditions. One proposed mechanism is that these variants alter DNA regulatory elements that govern the expression of genes involved in brain development, particularly during prenatal stages. Regulatory sequences active in the human fetal cortex have been found to be enriched with variants linked to traits such as intracranial volume, schizophrenia, attention-deficit/hyperactivity disorder (ADHD), depression, neuroticism, and educational achievement [46]. In addition, rare non-coding SNPs have been identified within regulatory regions that may influence genes related to ASD risk and genes expressed in the developing brain [48]. In a collective manner, these findings suggest that the impact of certain cognitive-trait-associated variants may arise through altered gene expression during early neurodevelopment [69]. An important complementary observation is that regulatory SNPs linked to cognitive phenotypes are often located in human-specific brain enhancers, many of which show signatures of positive selection [46,67,70].

Although GWAS have previously been applied to personality, the relationship between genome-wide transcriptional activity and personality traits in humans has only recently been investigated. Del Val et al. [71], using data from 459 participants in the Young Finns Study (ages 34–49), examined the regulation of gene expression and function associated with personality. Their analysis revealed two major gene regulatory networks: an extrinsic network of 45 regulatory genes originating from seed genes expressed in brain regions, including the basomedial amygdala, dentate nucleus, parahippocampal gyrus, and middle temporal gyrus, which are involved in the self-regulation of emotional reactivity to external stimuli (e.g., regulation of anxiety), and an intrinsic network of 43 regulatory genes derived from seed genes expressed in brain regions, including the lateral thalamic nuclei, angular gyrus, middle temporal gyrus, and cochlear nuclei, which are responsible for self-regulation of interpretive processes, such as concept formation and language. These two networks were found to be interconnected through a central hub composed of three microRNAs and three protein-coding genes. Interactions between this hub and various proteins and non-coding RNAs mapped directly onto more than 100 genes previously implicated in personality, as well as indirectly onto over 4000 additional genes. Based on these results, the authors argued that personality-related gene expression networks contribute to neuronal plasticity, epigenetic regulation, and adaptive responses by integrating processes of salience and meaning in self-awareness (e.g., insight and judgment). These findings are consistent with those of Zwir et al. [72], who demonstrated that resting-state functional connectivity (rsFC) of the prefrontal cortex provides stable, trait-like indicators of individual differences in perceptual, cognitive, emotional, and social domains.

## 3. Genetics of Substance Use Disorders

Since recruiting sufficiently large samples of individuals diagnosed with SUDs remains challenging, many genetic studies have instead examined broader use-related phenotypes such as initiation, frequency, or quantity of consumption, which can be more readily assessed in population-scale cohorts [73,74]. For instance, Saunders et al. [75] carried out one of the largest GWAS to date, involving nearly 3.3 million participants, and analyzed four tobacco-related traits (i.e., smoking initiation, age at onset of regular smoking, smoking cessation, and cigarettes smoked per day) along with alcohol consumption measured as “drinks per week”. This investigation identified an exceptionally high number of risk loci, including 1346 loci for smoking initiation and 496 for alcohol use. Regarding cannabis, the largest GWAS to date examined lifetime use [76], reporting eight genome-wide significant SNPs and implicating the genes *CADM2*, *SDK1*, *ZNF704*, *NCAH1*, *RABEP2*, *ATP201*, and *SMG6*. Another GWAS focusing on age at first cannabis use detected a single significant locus, which was *ATP2C2* [77].

Substance use traits show a moderate degree of genetic overlap with dependence and disorder phenotypes, pointing to a substantial shared biological basis. This suggests that GWAS of use-related phenotypes can provide meaningful insights into the etiology of SUDs. At the same time, the incomplete overlap highlights an important distinction between patterns of use and the development of dependence, reinforcing the need for GWAS that focus on rigorously defined SUD diagnoses in order to disentangle the specific mechanisms underlying substance dependence [78]. Moreover, compared to dependence phenotypes, use-based traits generally display weaker genetic correlations with psychiatric disorders and related characteristics. For example, alcohol use disorder (AUD) has been shown to correlate positively with ADHD and major depressive disorder (MDD), whereas alcohol consumption itself is negatively correlated with both [79]. A similar pattern can be noted with tobacco, in which phenotypes based on use exhibit much lower genetic correlations with psychiatric conditions than tobacco use disorder (TUD) [73,80].

Genetic effect sizes identified through GWAS can be aggregated into polygenic scores (PGS), which provide an estimate of the inherited liability of an individual for a given trait or disorder [81]. PGS serve multiple purposes, including validating GWAS findings, assessing genetic correlations with other traits, and probing gene–environment interactions. Although such scores generally capture only a modest proportion of trait variance, they are valuable for estimating individual-level genetic risk, making them an important component of predictive and analytical models [82]. The utility of PGS in predicting complex behavioral and psychiatric traits has been demonstrated across a range of phenotypes [83]. In the context of SUDs, current PGS explain approximately 2.1% of the variance in AUD [84], 3.8% in OUD [85], and 6.3% in TUD [80]. These predictive values remain lower than those reported for several other psychiatric disorders, largely reflecting the comparatively larger GWAS sample sizes available for those conditions [86].

Most studies indicate that PGS for AUD reliably predict both AUD itself and related alcohol consumption phenotypes [87,88,89], although a small number of analyses have failed to observe significant associations [90,91]. AUD PGS have been linked to earlier initiation of substance use, earlier onset of regular alcohol consumption, the emergence of alcohol-related problems, and formal diagnoses of alcohol dependence [92]. In addition, these scores are positively correlated with the use of other substances [84,93], and also with a range of psychiatric conditions, including depression, anxiety disorders, BD, ADHD, and pathological gambling [84,94]. Associations have also been observed with behavioral traits such as impulsivity [95] and resilience [96]. By contrast, AUD PGS have been shown to have negative associations with cognitive performance [93,97].

Research examining PGS for cannabis use disorder (CUD) remains limited. Segura et al. [98] reported that CUD PGS were significantly associated with cannabis use and monthly consumption at baseline, but not with age at first use or with measures related to the clinical trajectory following a first-episode psychosis. Conversely, Cheng et al. [99] found that CUD PGS predicted BD with psychotic features, whereas no association was observed for BD without them. In a related study, Paul et al. [100] explored the link between polygenic risk for substance use and cognitive performance. They found no significant relationship between CUD PGS and any cognitive measures. However, a PGS for lifetime cannabis use showed positive associations with general cognitive ability, executive functioning, and learning and memory.

Findings regarding PGS for other SUDs have been mixed. Two studies reported that OUD PGS significantly predict opioid use phenotypes [93,101]. In addition, OUD PGS showed positive correlations with a range of other substance use-related traits, while negative correlations have been observed with educational attainment and measures related to socioeconomic status. Positive associations have also been reported between OUD PGS and several mental health traits, including phenotypes related to conduct disorder and depression [93]. Nevertheless, Hartwell et al. [101] did not find significant relationships between OUD PGS and a variety of health-related phenotypes. In turn, Vilar-Ribó et al. [94] investigated the relationship between polygenic liability for five SUD-related phenotypes and ADHD. Their results indicated that PGS for cocaine dependence and a history of illicit drug addiction were not significantly associated with ADHD, whereas PGS for lifetime cannabis use, alcohol dependence, and smoking initiation were significantly correlated with ADHD. In a complementary approach, Hatoum et al. [102] derived a latent general addiction risk factor and demonstrated that PGS based on this factor were associated with SUDs and also with psychopathologies, somatic conditions, and environmental factors linked to addiction onset.

### 3.1. Specific Molecular Genetic Targets and Substances of Abuse

#### 3.1.1. Alcohol Use Disorder

The identification of consistently replicable genetic loci for AUD has been largely limited, with the notable exception of genes encoding alcohol-metabolizing enzymes, such as alcohol dehydrogenase 1B (*ADH1B*) and aldehyde dehydrogenase 2 (*ALDH2*) [103]. Recent investigations have advanced the detection of loci associated with AUD and related alcohol phenotypes [104,105]. In particular, genome-wide significant associations for *ADH1B* variants rs1229984 and rs2066702 with AUD have been consistently replicated [84,106,107,108], as well as with multiple alcohol consumption measures [73,79,106,109,110,111]. Comparable findings have been reported for *ALDH2*, specifically the rs671 variant, which shows robust associations with alcohol dependence and alcohol-related traits, including maximum drinks and flushing response, particularly in East Asian populations [112,113]. Furthermore, associations have also been observed for alcohol drinking status in these populations [114].

Recent GWAS have consistently identified links between genetic variants in the *DRD2* (dopamine receptor D2) gene and AUD, including rs4936277 and rs61902812 [106], as well as problematic alcohol use (PAU), with rs138084129 and rs6589386 [84,108]. Gene-based analyses have also associated *DRD2* with alcohol-related problems as measured by Alcohol Use Disorders Identification Test (AUDIT) scores [79,111]. Variants in the *GCKR* gene (rs1260326) have similarly been linked to AUD, alcohol use problems, and general alcohol consumption [73,79,84,106,108,109,111]. In addition, a Klotho Beta (*KLB*) variant rs13129401 has been associated with both PAU [108] and AUDIT-based measures of alcohol problems, as well as alcohol consumption [79,106,111]. Variants in the *SLC39A8* gene (solute carrier family 39 member 8) have also been linked to AUD (rs13107325) [106], alcohol problems (rs13135092) [79,111], and alcohol consumption (rs13107325) [106].

In summary, it should be noted that *ADH1B* and *ALDH2* genes have a direct influence on alcohol consumption, thereby modulating the risk for developing AUD. Coding variants in these genes confer a protective effect against AUD by eliciting aversive physiological responses to alcohol, which typically result in reduced consumption and lower disorder risk [103]. Nevertheless, it is likely that thousands of additional loci contribute to AUD susceptibility beyond those involved in alcohol metabolism. Recent studies examining subdomains of alcohol consumption suggest potential etiological distinctions between drinking frequency and quantity [115,116]. Specifically, consumption quantity shows greater genetic overlap with AUD and broader psychopathology, whereas drinking frequency exhibits negative associations with AUD and other psychiatric outcomes and appears to be influenced by socioeconomic factors [115,116].

#### 3.1.2. Cannabis Use Disorder

GWAS for CUD have yielded fewer replicable loci, primarily due to limited sample sizes [117]. To date, two genome-wide significant loci have been identified in humans. The first is located on chromosome 7 near the *FOXP2* gene (lead SNP: rs7783012), and the second on chromosome 8, encompassing brain expression quantitative trait loci (eQTLs) for *CHRNA2* and *EPHX2* (lead SNP: rs4732724) [118]. FOXP2 is involved in synaptic plasticity and has been implicated in speech and language development. Moreover, the risk variant rs7783012 has also been associated with externalizing behaviors [119]. *CHRNA2*, which encodes the α-2 subunit of the neuronal nicotinic acetylcholine receptor, has been implicated in prior GWAS of CUD [120], as well as in tobacco use and schizophrenia, both of which are phenotypically and genetically correlated with CUD [118]. *EPHX2* may contribute to cannabinoid metabolism, making it a plausible candidate for CUD, although it remains unclear whether *EPHX2* or *CHRNA2* is the causal driver of the association at this locus [117]. Another genome-wide significant variant, rs77378271 in the *CSMD1* gene, has been linked to both schizophrenia and the severity of cannabis dependence [118,121], but this association has not yet been replicated in additional CUD GWAS.

Evidence stemming from twin and family studies, together with GWAS of CUD, indicates substantial genetic overlap between CUD and other SUDs. CUD exhibits significant positive genetic correlations with smoking initiation, nicotine dependence, cigarettes per day, drinks per week, and AUD, with genetic correlations (rg) ranging from 0.31 to 0.66 [118]. Consistent with patterns observed for alcohol, recent GWAS highlight a distinction between cannabis use and CUD, both in terms of specific risk loci and broader genetic relationships with other traits and disorders. For instance, lifetime cannabis use (ever-use) is positively genetically correlated with educational attainment and age at first birth, and negatively correlated with body mass index (BMI) [76]. In contrast, CUD exhibits opposite genetic correlations for these traits [118], suggesting that the genetic basis underlying cannabis initiation is at least partially distinct from that contributing to CUD.

#### 3.1.3. Opioid Use Disorder

GWAS of OUD have identified significant loci near genes such as *KCNG2*, *KCNC1*, *APBB2*, *CNIH3*, *RGMA*, and *OPRM1* [122,123,124,125]. The largest GWAS of OUD, comprising 114,759 participants (15,756 cases), detected a functional coding variant in *OPRM1* (rs1799971) that reached genome-wide significance [108]. OUD also shows positive genetic correlations with other substance use traits, such as ever having smoked and alcohol dependence, as well as psychiatric disorders including ADHD and schizophrenia [108].

Although fewer studies have examined differences in the genetic etiology of OUD versus lifetime opioid use or non-dependent opioid use, evidence from the Psychiatric Genomics Consortium (PGC) suggests notable distinctions. Comparisons among opioid-dependent individuals, opioid-exposed controls, and opioid-unexposed controls revealed significant associations between a PGS for risk-taking and both contrasts of opioid dependence versus unexposed controls and opioid-exposed versus unexposed controls. A neuroticism PGS was associated with opioid dependence but not with the exposed versus unexposed control contrast, supporting the hypothesis that neuroticism contributes specifically to negative affect related to dependence rather than mere opioid exposure [125].

#### 3.1.4. Tobacco Use Disorder

Large-scale GWAS of nicotine dependence (ND) have consistently identified genome-wide significant associations with the cholinergic nicotinic receptor gene cluster *CHRNA5-A3-B4* on chromosome 15 [126]. These studies also revealed a novel association with an intronic variant (rs910083) in the *DNMT3B* gene, located on chromosome 20, which was subsequently linked to heavy smoking in the UK Biobank and implicated in lung cancer risk. Additional research from the Nicotine Dependence GenOmics Consortium further supported the association of a top variant in *CHRNA5* (rs16969968) on chromosome 15, and identified a genome-wide significant variant in *CHRNA4* (rs151176846) on chromosome 20 [127,128].

Extensive GWAS have also explored the genetic basis of additional nicotine-related traits [73]. For instance, a study identified 467 genome-wide significant loci across diverse smoking behaviors, including smoking initiation, cigarettes per day, smoking cessation, and age of onset for regular smoking [73]. Among single-variant associations, the strongest and most consistent finding was for the cigarettes-per-day phenotype, which showed robust association with rs16969968 in *CHRNA5*, replicating earlier results from independent cohorts [127,128,129]. Importantly, different smoking phenotypes display distinct genetic overlap with TUD. For instance, smoking initiation demonstrated only a moderate genetic correlation with ND (rg = 0.40), whereas the number of cigarettes smoked per day was almost perfectly correlated with ND (rg = 0.95). These results highlight that smoking initiation share less genetic liability with TUD compared to measures of smoking intensity (quantity of cigarettes per day) [128].

### 3.2. Genetic Epidemiology of Substance Use Disorders

Twin and family studies provide strong evidence for familial transmission of SUDs [130]. Across SUDs, heritability (h^2^) estimates generally indicate that genetic factors account for approximately 50% of individual risk. For AUD, heritability estimates are around 0.50 [131]. Estimates for AUD diagnosis are slightly higher than those for alcohol-related behaviors such as initiation (h^2^ ≈ 0.37) [132] and frequency of use (h^2^ = 0.37–0.50) [133]. This pattern aligns with prior twin research suggesting that environmental factors have a stronger influence on initiation, whereas genetic factors play a more prominent role in progression to heavier use and the development of alcohol-related problems [117].

Heritable influences are evident across stages of cigarette use and TUD, with heritability estimates for nicotine dependence ranging from 0.30 to 0.70 [134,135]. Variation in TUD heritability estimates may partially reflect differences in how smoking-related traits and problems are assessed [117]. For CUD, twin studies suggest heritability estimates between 0.48 and 0.51 [136], slightly exceeding those for cannabis use or initiation (h^2^ = 0.30–0.50) [137]. Shared genetic and environmental factors influence the progression from cannabis use to abuse. For instance, Gillespie et al. [138] found that cannabis availability accounted for nearly all shared environmental variance in both initiation and abuse, with initiation mediating the effect of availability on abuse, and 62% of the genetic variance in abuse overlapping with initiation. In turn, for opioid dependence, twin and family studies estimate that approximately 50% of liability is attributable to additive genetic factors [139]. In this respect, Mistry et al. [140] reported that 34% of the variance in opioid addiction is due to opioid-specific genetic influences. Table 1 summarizes the genes and variants associated with SUDs, key molecular genetic findings, and insights from genetic epidemiology studies of SUDs.

### 3.3. Genetic Approaches to Causality in Substance Use Disorders

For many critical health questions, conducting randomized controlled trials (RCTs) is often infeasible due to logistical or ethical constraints, limiting the ability to draw causal inferences. Advances in the genetics of substance use have enabled the use of the novel approach Mendelian randomization (MR) to address these challenges. In MR, genetic variants robustly associated with a putative risk factor, as identified through GWAS, are employed as instrumental variables [144,145]. This approach relies on three key assumptions: (i) the genetic variant must be strongly associated with the exposure; (ii) it must not be linked to confounders of the exposure–outcome relationship; and (iii) it must influence the outcome exclusively through the exposure pathway [146]. A notable limitation of conventional MR is its vulnerability to biases arising from assortative mating, dynastic effects, and population structure [147]. Such biases can be mitigated by utilizing family-based GWAS estimates in combination with standard MR techniques [147,148], or by applying within-family MR methods specifically designed to account for these confounding influences [149].

MR has been employed to investigate potential causal links between SUDs and various outcomes, including mental health, behavioral traits, and physical health measures [78]. Key traits studied include cognitive performance, educational attainment, structural brain measures, and psychiatric disorders such as MDD, post-traumatic stress disorder (PTSD), and ADHD. Although evidence consistently suggests that higher intelligence and greater educational attainment causally reduce the risk of developing AUD [84,150], the findings are not universally consistent. For instance, MR studies have not found causal effects in either direction between AUD and executive functioning [151] or between alcohol dependence and late-onset Alzheimer’s disease [152]. Similarly, there is an absence of compelling evidence that AUD causally influences psychiatric traits such as loneliness [153], self-harm [154], or suicide [155]. Moreover, despite some studies report a causal effect of ADHD on AUD [156], this finding has not been consistently replicated [94]. In contrast, there is stronger evidence that PTSD [157] and MDD [158] exert causal effects on AUD, whereas the reverse causal relationships appear unsupported.

Current evidence indicates bidirectional causal effects of educational attainment on CUD [159], whereas no causal relationship has been established between CUD and suicide [152]. There is evidence suggesting a causal effect of CUD on schizophrenia [160], but bidirectional influences cannot be fully excluded [161]. Regarding tobacco use and TUD, higher intelligence appears to reduce the risk of developing ND [162], while ND may increase susceptibility to schizophrenia [160]. In contrast, current data do not provide strong support for causal relationships between ND and ADHD [163] or between ND and suicide [155]. Similarly, there is no compelling evidence for a causal link between opioid dependence and suicide [155]. However, MDD and higher neuroticism have been shown to increase the risk of opioid dependence, whereas greater educational attainment appears protective [84]. Furthermore, in the context of cocaine dependence, Vilar-Ribó et al. [94] reported a lack of evidence supporting a causal association with ADHD.

## 4. The Roles of Genes and Personality in Addiction

Both genetic factors and personality traits have been recognized as key contributors to addiction susceptibility, together accounting for approximately 40–60% of the risk [164]. Certain personality characteristics, including high levels of adventurousness and propensity for risk-taking, are associated with an elevated likelihood of engaging in substance use [165]. Genetic differences, in turn, can influence how individuals respond to drugs, how quickly they metabolize substances, and their sensitivity to addictive effects. Variants in genes involved in dopamine signaling, such as the *DRD2* gene, are linked to reward deficiency, leading to higher risk behaviors [166]. This diminished sensitivity may drive a need for more intense or novel experiences to achieve rewarding effects, potentially increasing the probability of experimenting with addictive substances and progressing toward SUDs [166]. Similarly, genetic factors also influence the metabolism of alcohol, with enzymes encoded by *ADH1B* and *ALDH2* impacting alcohol dependence [143]. However, it is important to note that, in addition to the initiation of addictive behaviors, the dopaminergic system also contributes to the maintenance of addiction, reinforcing the compulsive seeking of substance over time [6]. In clinical terms, the challenges arising from addiction are distinct from the initial phases of use and require targeted interventions that address both the physiological and psychological aspects of addiction. In this context, early intervention could be crucial, as modifying the dopaminergic pathways at an early stage may prevent the escalation of risky behaviors. Nevertheless, implementing such interventions at a societal level can constitute a significant challenge, due to the complexities of widespread access, limited resources, and the need for large-scale behavioral and policy changes that can address the underlying social and environmental factors contributing to addiction.

Addiction and mental health disorders frequently co-occur, highlighting a strong interrelationship between both phenomena. Individuals experiencing conditions such as depression or anxiety, or those who struggle with social functioning, may use substances as a means of coping with distressing symptoms or emotions [167]. Moreover, people with obsessive-compulsive personality traits may be particularly vulnerable to developing SUDs due to persistent compulsions to consume psychoactive substances over time [168]. Indeed, such repetitive behaviors could reinforce patterns of drug use, potentially escalating into dependence or consolidated addiction [169].

Davis and Loxton [170] proposed that brain reward systems influence addiction risk primarily through their impact on the development of relatively stable personality traits associated with addictive behaviors. Several genes have been shown to modulate brain functions such as dopamine regulation and impulse control [171]. Novelty-seeking, impulsivity, and stress responsiveness, which constitute personality traits that are themselves partially heritable, seem to contribute to increased vulnerability to addictive behaviors [12,172]. However, there is no single “addiction-related gene”. Instead, numerous genetic variants can interact with environmental factors, particularly during adolescence, to shape the likelihood of developing an addiction. In this context, Teh et al. [173] hypothesized that variation in the dopamine D2 receptor (*DRD2*) gene may elevate addiction risk and severity. Their study of intravenous heroin users demonstrated a significantly higher prevalence of the *TaqIA* polymorphism among individuals with SUDs (69.9%) compared to controls (42.6%). Furthermore, affected individuals exhibited higher scores in novelty-seeking and harm-avoidance traits, but lower scores in reward dependence compared to controls. Additional research has highlighted the role of common gene variants, such as FTO and *TaqIA* rs1800497, in mediating gene-environment interactions that influence *DRD2* signaling, potentially promoting obesity, metabolic dysfunction, and cognitive alterations [174].

Zilberman et al. [2] suggested that the observed heterogeneity across different types of addiction may reflect underlying differences in personality traits specific to each addiction. In their study, the authors compared personality profiles across substance-related addictions (including drugs and alcohol) and behavioral addictions (such as gambling and compulsive sexual behavior). The sample comprised 216 individuals with SUDs and 78 control participants without a history of addiction. The results revealed distinct personality patterns across addiction types. Elevated impulsivity and neuroticism were observed across all addiction groups relative to controls. In contrast, individuals with AUD displayed lower levels of extraversion, agreeableness, and openness to experience. Notably, participants with SUDs and those with compulsive sexual behavior exhibited similar profiles, characterized by the lowest levels of agreeableness and conscientiousness. Interestingly, individuals with gambling disorder demonstrated a personality profile largely resembling that of the control group. Furthermore, personality traits were found to correlate with demographic variables, including socioeconomic status and religiosity. These findings support the hypothesis that personality traits may help distinguish between different forms of addiction. The study suggests that variations in personality development may contribute, at least in part, to the emergence of distinct addictive behaviors, providing a potential framework for understanding why individuals are prone to specific types of addiction.

Impulsive personality traits (IPTs) are heritable characteristics regulated by frontal-subcortical circuits and modulated by monoamine neurotransmitters, particularly dopamine and serotonin [175]. IPTs have been consistently linked to neuropsychiatric conditions, especially SUDs [176]. In a large-scale investigation, Sanchez-Roige et al. [111] conducted ten GWASs examining IPTs and drug experimentation in up to 22,861 adults of European ancestry. The study reported SNP heritabilities for IPTs and drug experimentation ranging from 5% to 11%. Notably, variants within the *CADM2* locus were significantly associated with UPPS-P Sensation Seeking and showed suggestive associations with drug experimentation. In addition, variants in the *CACNA1I* locus were significantly linked to UPPS-P Negative Urgency. These findings were supported by analyses at the single-variant, gene-based, and transcriptome-based levels. Furthermore, multiple subscales from the UPPS-P and Barratt Impulsiveness Scale (BIS) demonstrated strong genetic correlations with drug experimentation and other substance use phenotypes assessed in independent cohorts, including smoking initiation and lifetime cannabis use.

Dash et al. [177] reported that familial influences on personality traits and substance use are somewhat generalized. Specifically, higher levels of neuroticism and openness to experience, along with lower agreeableness, were associated with the use of multiple drug types. Elevated neuroticism was particularly linked to prescription drug misuse. Conversely, higher extraversion correlated with cocaine, crack, and stimulant use. In turn, greater openness to experience was associated with cannabis consumption. Lower agreeableness showed associations with both cocaine/crack and illicit opioid use. Notably, no within-pair effects were detected for conscientiousness, suggesting that this trait may play a less direct role in familial risk for substance use.

GWAS of SUDs, including problematic use of tobacco, alcohol, cannabis, and opioids, have highlighted a component of genetic liability that is shared across these disorders. To investigate this shared risk, Hatoum et al. [102] conducted multivariate GWAS combining datasets for AUD, TUD, CUD, and OUD, encompassing a total sample of over one million individuals. Using genomic structural equation modeling, the authors identified a general addiction risk factor associated with 17 independent loci reaching genome-wide significance. Gene-based analyses further revealed significant associations with 42 genes, including *FTO*, *DRD2*, and *PDE4B*. Moreover, linkage disequilibrium score regression indicated positive genetic correlations between this general addiction risk factor and traits such as suicide attempt, self-medication for anxiety or depression, and externalizing behaviors.

Maciocha et al. [178] investigated the relationship between the microsatellite polymorphism (AAT)n in the Cannabinoid Receptor 1 (*CNR1*) gene and personality traits in women with AUD. The study included 93 female participants diagnosed with AUD and 94 control subjects. Compared to controls, women with AUD scored significantly higher on both the State-Trait Anxiety Inventory (STAI) state and trait scales, as well as on the Neuroticism and Openness scales of the NEO Five-Factor Inventory (NEO-FFI). Conversely, the AUD group exhibited lower scores on the NEO-FFI Extraversion, Agreeableness, and Conscientiousness scales. In addition, no statistically significant Pearson correlations were observed between the number of (AAT)n repeats in the *CNR1* gene and STAI or NEO-FFI scores within the AUD group. However, in the control group, the number of (AAT)n repeats showed a positive correlation with the STAI state scale and a negative correlation with NEO-FFI Openness. The study highlighted two main conclusions: (i) a potential association of (AAT)n *CNR1* repeats with AUD in women, and (ii) a link between (AAT)n *CNR1* repeats and both state anxiety and Openness in individuals without AUD.

Gambling disorder (GD) is characterized by persistent, harmful, and recurrent engagement in gambling-related behaviors and shares biological mechanisms and symptomatology with SUDs. Recław et al. [179] examined the association between the *COMT* gene polymorphism and behavioral addiction. The study included 307 male participants: 107 individuals diagnosed with GD and amphetamine use disorder, and 200 non-addicted controls without neuropsychiatric disorders. Both groups completed psychometric assessments using the STAI and the NEO-FFI. Compared to controls, participants with GD and amphetamine use disorder scored higher on the STAI state and trait scales as well as the NEO-FFI Neuroticism scale. Conversely, they exhibited lower scores on the NEO-FFI Agreeableness scale. Furthermore, a significant interaction was observed between the presence of GD or amphetamine use disorder and the *COMT* rs4680 genotype on STAI state and trait scores, as well as NEO-FFI Conscientiousness scores. Thus, the findings obtained suggest that the *COMT* gene and its polymorphic variants may contribute to the development of addictive behaviors.

## 5. Epigenetic Influences on Addiction

Environmental influences and mental health conditions play a pivotal role in shaping the onset and trajectory of addiction. Indeed, individuals experiencing certain psychiatric disorders are particularly susceptible to developing SUDs, often as a means of self-medicating or temporarily alleviating emotional distress, which can create a reinforcing cycle leading to addiction [9]. In this context, peer groups that normalize or encourage substance use further increase the likelihood of addictive behaviors. Certainly, the impact of environmental factors on addiction is substantial. A meta-analysis reported an effect size of 0.61 for environmental influences, indicating a stronger relationship with addiction risk compared to individual factors, which had an effect size of 0.45 [180]. Moreover, ACEs can leave enduring emotional scars, increasing the propensity to use substances as a coping mechanism for unresolved trauma [17]. Nevertheless, the influence of environmental factors on addiction outcomes is modified by the genotype of individuals, highlighting the complex, multifactorial nature of addiction vulnerability [181].

Epigenetics examines how environmental factors can influence gene expression without altering the underlying DNA sequence. This regulatory system operates through multiple mechanisms, including DNA methylation, histone modifications, non-coding RNA (ncRNA) regulation, RNA modifications, and chromatin remodeling [182,183]. DNA methylation is a central epigenetic mechanism that modulates gene expression by adding methyl groups to cytosine residues, often within CpG islands. This process can inhibit gene transcription by preventing transcription factor binding or recruiting repressive proteins, such as methyl-CpG-binding domain proteins [184]. Histone modifications are also key regulators of chromatin accessibility and gene activity. Depending on the type of modification and the specific amino acid residue involved, these changes can either activate or repress gene expression [185]. Common histone modifications include methylation of lysine and arginine residues, acetylation of lysines, phosphorylation of serine, threonine, or tyrosine, ubiquitination of lysines, and less frequent modifications such as SUMOylation, ADP-ribosylation, deamination, and proline isomerization [186]. Among these, histone methylation and acetylation have been most extensively studied. Histone methylation, mediated by histone methyltransferases and demethylases, can either repress (e.g., H3K27me3) or promote (e.g., H3K4me3) transcription. Conversely, histone acetylation, controlled by histone acetyltransferases (HATs) and deacetylases (HDACs), generally leads to chromatin decondensation and enhanced transcriptional activity [187]. In addition, ncRNAs are another critical layer of epigenetic regulation. Major classes of ncRNAs include microRNAs (miRNAs), long ncRNAs (lncRNAs), and circular RNAs (circRNAs). MiRNAs modulate gene expression by binding to the 3′ untranslated regions (3′ UTRs) of target mRNAs, resulting in translational repression or mRNA degradation [188].

Research indicates that environmental exposures such as drug use, trauma, and chronic stress can modulate gene expression, including genes implicated in addiction [189]. Prolonged stress, for instance, can alter regulatory mechanisms within the brain’s reward circuitry, thereby heightening vulnerability to SUDs. Similarly, repeated exposure to drugs can induce gene expression changes that reinforce compulsive behaviors, increasing the risk of persistent substance abuse. Markunas et al. [190] conducted the first epigenome-wide association study (EWAS) of smoking in human post-mortem brain tissue, focusing on the NAc. They identified seven DNA methylation (DNAm) biomarkers: three corresponded to genes previously implicated as blood-based DNAm biomarkers of smoking, and four were novel, including *ABLIM3*, *APCDD1L*, *MTMR6*, and *CTCF*. In the context of AUD, Lohoff et al. [191] performed DNAm EWAS analyses to identify epigenetic modifications relevant to this condition. They found networks of differentially methylated regions in genes related to glucocorticoid signaling and inflammatory pathways. A prominent probe consistently associated across cohorts was located within the long non-coding RNA GAS5, which showed elevated expression in the amygdala of individuals with AUD. Subsequent analyses revealed that *SLC7A11*, encoding the cystine-glutamate antiporter, was overexpressed in the frontal cortex and liver of individuals with AUD, suggesting a mechanism in which alcohol-induced hypomethylation drives overexpression of this gene [192]. Regarding cannabis use, Fang et al. [193] conducted a peripheral blood-based DNAm EWAS meta-analysis of lifetime cannabis use (ever vs. never) across seven cohorts totaling 9436 participants (7795 European ancestry and 1641 African ancestry). After controlling for cigarette smoking, four CpG sites were significantly associated with cannabis use: cg22572071 near *ADGRF1*, cg15280358 in *ADAM12*, cg00813162 in *ACTN1*, and cg01101459 near *LINC01132*. Furthermore, in participants who never smoked cigarettes, an epigenome-wide significant CpG site, cg14237301 annotated to *APOBR*, was identified.

### 5.1. The Role of the Gut Microbiome

**Figure 1:**
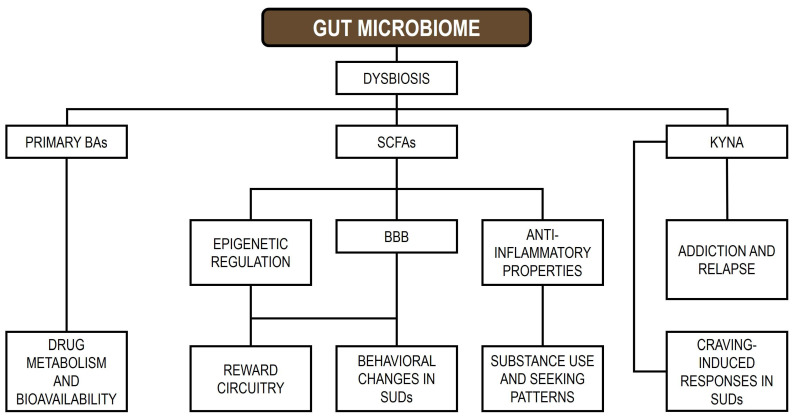
Signal pathways between GM metabolites and SUDs. BAs: bile acids; SCFAs: short-chain fatty acids; KYNA: kynurenic acid; BBB: blood–brain barrier; SUDs: substance use disorders.

The GM contributes to addiction by producing neurotransmitters and metabolites that communicate with the GBA, modulating the expression of genes involved in reward circuitry, including those regulating dopamine signaling, and promoting pro-inflammatory states [38]. Host genetic variation can shape the composition of the GM, and disturbances in microbial communities may alter gene expression, potentially increasing susceptibility to addictive behaviors [194]. The GM affects host gene regulation through the secretion of metabolites such as SCFAs, tryptophan derivatives, and BAs, which interact directly with host cells, influence BBB permeability, and modulate neural signaling, thereby impacting gene expression and behavior [195]. Moreover, the GM produces neurotransmitters, including serotonin, dopamine, and GABA, which can influence brain function and gene expression within reward-related neural circuits [196,197]. Interactions between the GM and host can also induce epigenetic changes, including DNA methylation, histone modifications, ncRNA regulation, and transcriptional alterations in host cells, ultimately affecting genes involved in immunity, metabolism, and gut barrier integrity [39,198]. Figure 1 illustrates the signaling pathways linking GM-derived metabolites to SUDs (modified from [37]).

In essence, the relationship between the host and the GM is bidirectional: host genetics and lifestyle factors, including substance use, shape the composition and function of the GM, which in turn communicates with the brain through multiple pathways, modulating gene expression and potentially increasing susceptibility to, or reinforcing, addictive behaviors [37]. This dynamic interaction appears to have a substantial impact on both gut and brain health [199]. Evidence indicates that substance abuse can induce GM dysbiosis, manifested as altered microbial diversity, disrupted community composition, and decreased levels of SCFAs [200]. In turn, mechanistic studies suggest that drug-induced dysbiosis may compromise gut barrier integrity and promote heightened local and systemic inflammatory responses. These changes can initiate a cascade of physiological and behavioral effects that exacerbate SUDs and contribute to the maintenance of addictive behaviors.

#### 5.1.1. Substance Use and Gut Microbiome Composition

A recent review highlighted the potential involvement of GM dysbiosis in the development of SUDs, indicating that alterations in the GM may influence addiction through modifications in GBA signaling [37]. Furthermore, changes in GM composition and metabolite profiles may not only be a consequence of SUDs but could also modulate behavioral responses to addictive substances. Regarding AUD, variations in the relative abundance of certain genera, such as *Faecalibacterium*, *Gemmiger*, *Escherichia*, and *Fusobacterium*, may serve as potential biomarkers for predicting cognitive impairments in domains including emotional processing, memory, and executive function [201]. Ling et al. [202] reported that AUD patients exhibited reduced levels of the butyrate-producing genera *Faecalibacterium* and *Gemmiger*, which positively correlated with cognitive performance [201] and negatively correlated with pro-inflammatory markers, including TNF-α and various chemokines [202]. In a related review, Chen et al. [41] observed that alcohol exposure is associated with an increased relative abundance of *Pseudomonadota*, *Enterobacteriaceae*, *Fusobacteriota*, *Clostridium*, and *Lactococcus*, alongside a decreased abundance of members of the phyla Bacillota and Bacteroidota. Similarly, various studies have shown that alcohol consumption increases the abundance of the bacterial genera *Clostridium*, *Holdemania*, and *Sutterella*, while reducing the abundance of *Akkermansia muciniphila* and *Faecalibacterium prausnitzii* [203,204].

Regarding cannabinoids, Vijay et al. [205] reported that the endocannabinoid system is positively associated with bacterial α-diversity and with butyrate- and SCFA-producing genera, including *Bifidobacterium*, *Coprococcus*, and *Faecalibacterium*, whereas negative associations were observed with *Collinsella* and *Escherichia/Shigella*. It has also been noted that cannabis use may induce shifts in GM composition, particularly affecting the *Prevotella*/*Bacteroides* ratio [206]. In addition, SCFAs produced by the GM exert anti-inflammatory effects and can epigenetically modulate gene expression, while alterations in kynurenic acid (KYNA) metabolism have been linked to decreased drug-seeking behaviors for substances such as cannabis [206].

There is a notable scarcity of clinical studies investigating the effects of tobacco use on the GM. Nevertheless, nicotine withdrawal has been linked to substantial alterations in GM composition, including increased microbial diversity and a higher relative abundance of the phyla Bacillota and Actinomycetota, accompanied by a reduction in Bacteroidota and Pseudomonadota members [207]. Shanahan et al. [208] reported that smokers exhibit lower bacterial diversity in the upper small intestinal mucosa compared to non-smokers. In smokers, the GM showed elevated levels of Bacillota (notably *Streptococcus* and *Veillonella*) and Actinomycetota (*Rothia*), along with decreased abundance of Bacteroidota (*Prevotella*) and Pseudomonadota (*Neisseria*). In contrast, Stewart et al. [209] observed an increase in Pseudomonadota and Bacteroidota, with predominant genera being *Clostridium* and *Prevotella*, while *Bacteroides* levels were reduced. In another study, Savin et al. [210] noted an increase in the abundance of the Bacteroidota and Pseudomonadota phyla, as well as in the genera *Bacteroides*, *Clostridium*, and *Prevotella*. Furthermore, they reported a decline in the abundance of the Actinomycetota and Bacillota phyla, as well as in the genera *Bifidobacterium* and *Lactococcus*.

Opioid use exerts profound effects on the gut by slowing peristalsis and inducing constipation, which can increase gut barrier permeability and promote bacterial translocation [200,211]. These disruptions contribute to GM dysbiosis, which in turn plays a role in opioid tolerance. Alterations in GM composition can exacerbate opioid effects, creating a detrimental positive feedback loop that further impairs gut health and opioid responsiveness [212]. In patients with OUD, the GM exhibits increased α-diversity, likely due to delayed colon transit that promotes bacterial proliferation within the gastrointestinal tract [213]. Chronic opioid users have been reported to show reduced abundance of the phylum Bacteroidota, the family *Bacteroidaceae*, and the genus *Bacteroides* [214,215]. Findings regarding other taxa, including *Prevotella*, *Bifidobacterium*, *Ruminococcus*, and the family *Ruminococcaceae*, have been inconsistent across studies [214,215,216]. Furthermore, opioid exposure has been associated with decreases in the family *Bacteroidaceae*, as well as in the genera *Lactobacillus* and *Bifidobacterium*, while simultaneously increasing the prevalence of potentially pathogenic genera such as *Enterococcus*, *Flavobacterium*, *Fusobacterium*, *Sutterella*, *Ruminococcus*, and *Clostridium* [57,217,218].

#### 5.1.2. Social and Microbial Influences on Substance Use Risk

Adolescence represents a critical developmental period marked by extensive changes in neuronal structure and function, which are related to the acquisition of behavioral and social skills [219,220]. This life stage also coincides with the consolidation of the GM [221]. Epidemiological evidence indicates that drug experimentation during adolescence increases the risk of developing SUDs, underscoring the heightened vulnerability of this developmental stage to substance-related effects [222,223,224]. In this respect, alterations in the GM during early life, combined with exposure to adolescent social stressors, can disrupt GBA signaling, potentially promoting neurodevelopmental changes that increase susceptibility to drug exposure [38]. Consistently, recent research indicates that adolescence is more sensitive than adulthood to the combined effects of cocaine exposure and early maternal deprivation, indicating that the accumulation of stress during early life can exacerbate the negative behavioral outcomes associated with substance use [225].

Early-life stress (ELS) is recognized as a factor capable of reshaping neural circuitry, modifying stress reactivity, and initiating neuroinflammatory processes that function as mechanisms in response to adversity [226]. Nevertheless, when inflammation becomes persistent, it can give rise to maladaptive outcomes such as sickness behavior, which is linked to anhedonia and social withdrawal [227]. This highlights the significant role of the immune system in modulating neuronal networks that regulate both social functioning and reward processing. Consequently, exposure to social stressors during early developmental stages can profoundly impact the central nervous system, leading to alterations in stress regulation, disruption of social interactions, and heightened vulnerability to SUDs [228].

In addition to neuroimmune mechanisms, emerging evidence points to the GM as another early-life determinant of long-term health. Insufficient or dysregulated microbial exposure during critical developmental stages may provoke inflammatory activity and has been implicated in a variety of physiological disturbances [229]. In this respect, ELS can impact cognitive function, with maternal early-life nutrition influencing the effects of prenatal and postnatal stress [230]. Moreover, ELS-induced alterations in the GM can disrupt the production of microbial metabolites and neurotransmitters, potentially modulating stress responses and neurodevelopmental trajectories [230]. Although associations between ELS and GM alterations have been reported during both prenatal and postnatal periods, a consistent microbiome profile specifically linked to stress exposure at these stages has yet to be fully elucidated [229].

Early-life adversities during childhood and adolescence (i.e., ACEs) are strongly associated with long-term consequences that persist into adulthood, including subsequent onset of SUDs [231,232]. In particular, bullying victimization during childhood has been identified as a significant risk factor for later substance use [233]. This may be partly explained by attempts to alleviate the adverse emotional states elicited by bullying, particularly humiliation, which has been associated with highly concerning outcomes, including psychopathology and suicide [234]. In preclinical research, the social defeat paradigm, which is commonly used to model bullying in rodents, has also been shown to enhance drug consumption in later life [235,236]. In addition, exposure to social defeat during adolescence heightens responsiveness to alcohol reward, a phenomenon that may involve dysregulation of the hypothalamic–pituitary–adrenal (HPA) axis within mesocorticolimbic circuits [237]. Importantly, the neurobehavioral outcomes of social stress are not uniform but instead vary according to individual differences in personality traits [238]. Consistent with this variability, adolescent social defeat has been linked to enhanced reinforcing effects of psychostimulants such as cocaine and to elevated levels of pro-inflammatory mediators, including interleukin-6 (IL-6) [239]. These cytokines are known to influence dopaminergic neurons in reward-related pathways, thereby modulating neurotransmission and synaptic plasticity [240,241], and also exerting profound effects on social behavior [227]. Furthermore, adolescent social isolation has been shown to increase susceptibility to SUDs in adulthood, likely through stress-related modifications in corticotropin-releasing hormone signaling and disruption of oxytocin system maturation within the NAc and paraventricular nucleus [242].

Substance use itself can function as a potent stressor, altering stress-regulatory systems and amplifying vulnerability to addiction when combined with other adverse conditions. The extent and nature of these effects depend on the pharmacological class of the substance and on patterns of consumption [243]. Acute drug exposure typically activates the stress response, leading to elevations in cortisol or corticosterone [244]. In contrast, chronic use induces more complex adaptations within the HPA axis, the adrenergic system, and the autonomic nervous system, reflecting the multifaceted ways in which drugs reshape stress physiology [245]. An often overlooked aspect of addiction is its strong association with social isolation and exclusion. Individuals with SUDs frequently withdraw from social interactions or are marginalized by their communities, making reintegration particularly challenging [246]. Such isolation may arise from stigma, fear, or social judgment. In fact, it has been noted that stigma linked to SUDs exerts detrimental effects across multiple domains, impeding treatment seeking, compromising the quality of professional care, influencing public policy decisions, and hindering social inclusion [247]. Stigma acts as a persistent psychosocial stressor, promoting adverse mental states such as anxiety and depression, which may subsequently trigger or aggravate substance use as a means of stress-related self-medication [248]. This bidirectional dynamic perpetuates both stigmatization and substance dependence, indicating a self-reinforcing cycle. Indeed, experiences of loneliness and social disconnection can serve as aversive states that drive individuals to use substances as a maladaptive coping strategy [249]. In this context, AUD has been identified as one of the most highly stigmatized substance-related conditions, despite alcohol being a legal and socially accepted substance with widespread global consumption, including among adolescents [250]. This reciprocal relationship between social stressors and drug use underscores the critical role of psychosocial factors in addiction escalation. Interventions aimed at reducing stigma and strengthening social bonds may therefore represent valuable strategies for mitigating dependence and promoting recovery.

From a microbial perspective, targeting the GM offers a promising pathway for interventions in drug reward–related disorders, either as a standalone strategy or in synergy with social factors. Recent findings by García-Cabrerizo et al. [251] demonstrate that depletion of the GM diminishes the reinforcing properties of non-natural rewards such as cocaine and simultaneously enhance the value of social rewards. Notably, when antibiotic-induced microbiota depletion was paired with the presence of social stimuli, cocaine preference was further reduced. Thus, these results suggest that a dual approach (i.e., modulating the GM and promoting social engagement) may represent a pivotal approach for mitigating the salience of drug-related cues and reducing drug-related harm [251].

#### 5.1.3. The Gut Microbiome and Personality Traits

Evidence suggests that GM dysbiosis may play an important role in the development of neuropsychiatric and psychological disorders [252]. A key unresolved question, however, is whether variation in microbial community composition contributes to stable personality traits and behavioral patterns that remain consistent across time and contexts, and which could be therefore predictable. Preclinical research has demonstrated that the GM can modulate stress reactivity, anxiety- and depression-like behaviors, as well as social interaction and communication [253]. Some of the most compelling data come from fecal microbiota transplantation (FMT) experiments, which reveal that behavioral phenotypes can be transferred between mouse strains through microbiota exchange [254]. For instance, colonization of typically anxious Balb/c mice with the microbiota of NIH Swiss mice induces a shift toward greater boldness and exploratory behavior, mirroring the donor phenotype, and the reverse also occur [255]. Additional evidence comes from rodents colonized with the microbiota of individuals with anxiety and depression, which subsequently exhibit corresponding behavioral disturbances [256]. Consequently, these findings suggest that gut microorganisms can exert a causal influence on behavioral traits.

Only a limited number of studies have examined the association between the GM and personality traits. Kim et al. [257] reported that both the diversity and composition of the human GM varied according to scores on the revised NEO Personality Inventory. Although the differences observed were subtle, significant correlations were identified between microbial diversity and specific personality dimensions. For instance, higher neuroticism and lower conscientiousness were linked to increased relative abundances of Pseudomonadia (formerly classified as Gammaproteobacteria) and Pseudomonadota, respectively. Conversely, individuals with higher conscientiousness exhibited greater abundance of certain butyrate-producing taxa, particularly members of the *Lachnospiraceae* family. Building on these findings, Johnson [258] investigated whether variation in GM diversity and composition could be predicted by individual differences in personality. Using negative binomial regression, the study showed that seven out of 23 bacterial genera were significantly associated with behavioral traits. Sociability, which is a composite measure including extraversion, social skill, and communicative ability, positively predicted the abundance of *Akkermansia*, *Lactococcus*, and *Oscillospira*, and was inversely related to *Desulfovibrio* and *Sutterella*. In contrast, neurotic tendencies, which is a combined index of neuroticism, anxiety, and stress, were negatively associated with *Corynebacterium* and *Streptococcus*. The study also demonstrated that anxiety- and stress-related traits were linked to altered microbial composition, and that the presence of a mental disorder significantly influenced GM profiles. Moreover, although exercise frequency did not predict microbial diversity, it was significantly associated with overall GM composition. More recently, Park et al. [259] identified lower microbial richness in individuals classified as high in anxiety and vulnerability compared with those scoring low on these traits. Significant differences in β-diversity were also observed across groups stratified by anxiety, self-consciousness, impulsivity, and vulnerability. In addition, the genus *Haemophilus* was associated with neuroticism, while reduced abundances of *Christensenellaceae* were observed in high-anxiety and high-vulnerability groups. Similarly, lower levels of *Alistipes* and *Sudoligranulum* were linked to elevated self-consciousness. Table 2 summarizes microbial associations with various personality traits across childhood and adulthood (modified from [260]).

## 6. Discussion

SUDs represent a group of highly prevalent and heritable psychiatric conditions that mainly arise from the interaction of genetic vulnerability and environmental influences. SUDs affect hundreds of millions of individuals worldwide and contribute substantially to the global burden of disease. In 2019, AUD affected more than 100 million individuals and was linked to approximately 160,000 deaths [274]. In turn, OUD affected about 21 million individuals and accounted for over 88,000 deaths [274]. In addition to their direct health consequences, SUDs also elevate the risk for major causes of morbidity and mortality, including suicide, self-harm, and medical conditions such as chronic obstructive pulmonary disease [78]. Despite their profound societal and clinical relevance, relatively little is known about the personality traits that may predispose individuals to substance use initiation, escalation, and eventual dependence. In this respect, the notion of an “addictive personality” has been inconsistently defined and debated, but several recurring assumptions have shaped its interpretation. First, it has been proposed that individuals who later develop addictions already possess a stable personality profile that predisposes them to substance use, characterized by traits such as impulsivity, sensation seeking, antisocial tendencies, and nonconformity. Second, the concept suggests that such individuals display predictable patterns of cognition and behavior, including persistent drug preoccupation, compulsive use despite adverse outcomes, and prioritization of substance use over other significant activities. Third, the framework assumes that, because of these enduring personality dynamics, individuals labeled as having an “addictive personality” are more likely to substitute one form of drug use or compulsive behavior for another during or after treatment [12,23,275,276]. These assumptions are problematic for several reasons. They contribute to pathologizing and stigmatizing narratives that portray individuals with SUDs as a homogeneous group, despite substantial heterogeneity in clinical presentation and etiology [277]. They also encourage negligent clinical practices in which pseudodiagnostic judgments are applied without empirical basis, thereby limiting the quality of care across medical, mental health, and social services. Finally, such labels may undermine the self-efficacy of individuals with SUDs, who are already marginalized and separated from other subjects facing health challenges [23]. Taken together, these considerations underscore that certain personality traits may influence susceptibility to substance use and to addiction, but the notion of a fixed “addictive personality” is simplistic and potentially misleading, as SUDs emerge from complex interactions between multiple factors.

Building on the aforementioned, although personality traits alone cannot determine the onset of addiction or susceptibility to substance use, it can be noted that certain characteristics appear with greater frequency among individuals with SUDs, particularly heritable traits such as impulsivity, novelty-seeking, and stress responsiveness. Impulsivity is characterized by diminished inhibitory control and a tendency toward rash decision-making, which is what could promote maladaptive patterns of substance use. This specific trait shapes how individuals respond to stress and seek rewarding experiences, thereby influencing susceptibility to addictive behaviors. Importantly, heightened sensitivity to novel and rewarding stimuli has been associated with sensation seeking and an increased likelihood of risk-taking [278]. Novelty-seeking may interact with impulsivity, predisposing individuals to behaviors that increase the risk of addiction. Thus, novelty-seeking may also be a trait highly relevant to substance use. Given that motivation plays a pivotal role in the onset and progression of SUDs, particularly when initial engagement with substances is driven by curiosity and the pursuit of novel sensations [18], it is plausible that individuals high in novelty-seeking and openness may exhibit a greater inclination toward drug experimentation. When such exploratory tendencies co-occur with elevated impulsivity and stress responsiveness, a synergistic pattern may emerge, in which novelty-seeking facilitates exposure to substances, impulsivity accelerates transition from experimentation to maladaptive use, and heightened stress responsiveness amplifies vulnerability under potentially challenging or adverse conditions. Furthermore, compulsivity may interact with these traits, reflecting the difficulty that individuals experience in resisting substances and contributing further to addiction susceptibility [279]. In fact, compulsive patterns may partly reflect heritable influences, suggesting that genetic factors could also contribute to compulsivity in substance use [280]. In this respect, it is essential to note that this compulsion should be understood as strong urges that are hard to resist, rather than behavior that is entirely involuntary [275]. Nevertheless, this configuration of traits does not constitute itself a sufficient condition for addiction. Instead, it may more likely represent a personality profile that confers heightened susceptibility to substance use under conducive environmental or contextual factors.

Extending the previous considerations, among the various neurocognitive functions associated with SUDs, the dimensions of impulsivity have received the strongest empirical support as potential endophenotypes [172,281]. Neurocognitive impulsivity can be subdivided into two primary dimensions, typically assessed through behavioral tasks [282]. First, decision or choice impulsivity reflects the tendency to favor immediate, smaller rewards over delayed, larger ones, capturing deficits in delayed gratification and self-control. This dimension is commonly evaluated using decision-making paradigms that manipulate risk, reward, and delay intervals [283,284]. Second, motor or action impulsivity pertains to difficulties in inhibiting inappropriate responses, typically measured through response inhibition tasks [285,286]. Notably, individuals addicted to different classes of substances, such as opioids versus stimulants, may exhibit distinct profiles across these impulsivity dimensions [287,288]. The endophenotypic approach, widely used in psychiatric genetics, offers a framework for bridging the gap between complex behavioral disorders and their underlying genetic basis. Endophenotypes are quantifiable, heritable traits that are more stable and less complex than clinical phenotypes, facilitating the identification of specific genetic variants, neural circuits, and environmental interactions that contribute to disease susceptibility and progression [289]. Recent conceptual developments suggest that focusing on endophenotypes may help reduce the heterogeneity of SUD presentations and provide a structured approach for distinguishing general versus substance-specific risk factors [290]. Thus, neurocognitive dimensions of impulsivity may serve as measurable endophenotypes that link genetic vulnerability to behavioral outcomes and enable differentiation of general from substance-specific risk pathways in addiction.

Alcaro et al. [6] have emphasized that identifying genes associated with addiction represents an essential first step toward developing targeted interventions. Several considerations underscore the importance of this approach. First, elucidating the biological mechanisms by which specific genes influence addiction can inform the development of more effective treatments for SUDs. Second, each newly identified addiction-related gene represents a potential pharmacological target, enabling researchers to design drugs that modulate the activity of the corresponding protein. Third, gene-based therapies are emerging as a promising avenue for addiction treatment. For instance, one experimental gene therapy in mice induces the production of antibodies that trap methamphetamine, thereby preventing its entry into the brain [291]. Similarly, transplantation of genetically engineered skin cells in mice has been used to produce enzymes capable of degrading cocaine [292]. Finally, in the long term, genetic profiling could allow personalized treatment strategies by predicting which interventions are most likely to be effective for an individual based on their unique genetic profile.

Experimental evidence increasingly supports a pivotal role for the GM in SUDs, acting through multiple mechanisms. These include modulation of gene expression within the NAc [293], regulation of reward and addiction circuits [294], and alterations in pain perception [295]. Such findings underscore the direct involvement of the GM in both substance-induced reward responses [296] and withdrawal states [166], highlighting its relevance across different stages of addiction. Interestingly, the GM can also metabolize substances of abuse, influencing their pharmacokinetics and efficacy, thereby modifying the intensity of reward and withdrawal experiences [34,297]. In addition to these effects, the GM contributes to epigenetic regulation by producing metabolites that modulate the epigenome and by influencing the expression of epigenetic enzymes. This regulatory capacity may also affect comorbid outcomes, including anxiety and depression, which are frequently associated with SUDs [198]. Conversely, chronic substance use induces stable epigenetic modifications that alter gene expression and behavior [187]. Future investigations integrating analyses of DNA methylation, histone acetylation, crotonylation, and other epigenetic marks with GM composition and activity of epigenetically active metabolites could elucidate the mechanisms by which the GM shapes addiction-related epigenetic changes. In this respect, recent work by Lewin-Epstein et al. [298] demonstrates that GM dysbiosis affects host brain function, behavior, and overall wellbeing, and is linked to the onset and progression of chronic disorders, including addictive behaviors. The authors propose that competitive interactions within the GM may drive evolutionarily relevant effects on host behavior, potentially contributing to addictive tendencies. Moreover, feedback from the GM to host behavior can exacerbate addictive patterns, complicating withdrawal and increasing relapse risk. Microbial richness appears to be a critical factor in this process, with lower richness associated with prolonged addiction trajectories.

The studies included in this review present several notable limitations. First, regarding genetic research on personality traits and SUDs, particularly AUD, only a small proportion of the underlying genetic factors has been identified. This “missing heritability” problem may arise from the complex interplay between genetic variants, environmental influences, and their interactions [178]. Despite large-scale GWAS, researchers have been able to explain only a fraction of trait variance. Contributing factors include the fact that certain genomic variants, such as copy number variants (CNVs), insertions/deletions, variable number tandem repeats (VNTRs), and short tandem repeats (STRs), are not typically assessed in GWAS, but they may significantly influence phenotypic variability [299,300]. Another limitation is the lack of diversity in GWAS samples. Most studies to date have been conducted predominantly in individuals of European ancestry, which limits the generalizability of findings to other populations and may exacerbate health disparities. Increasing representation of non-European ancestries is therefore critical for advancing understanding of SUD genetics. Large but unrepresentative cohorts, such as the UK Biobank, overrepresented by older individuals with higher socioeconomic status, or the Million Veteran Program (predominantly male), also pose challenges, including the potential for collider bias [301]. Additional sources of bias include misreporting, longitudinal changes in behavior [302], and the complex interplay between genetic and sociocultural factors in substance use and SUD development [116]. Deak and Johnson [117] emphasize that evaluating multiple measures of substance use and SUDs, including both clinical diagnoses and brief questionnaires across diverse sample types, is essential to disentangle the genetic basis of consumption versus problematic use. With respect to studies on the GM and SUDs, several limitations should also be noted: (i) confounding variables, as many studies fail to account for pre-existing mental health conditions; (ii) limited sample diversity, with restrictions in age or cultural background that reduce generalizability; (iii) cross-sectional study designs, which hinder causal inferences; (iv) self-reporting bias; (v) the influence of genetic and environmental factors on both GM composition and SUD outcomes; (vi) lack of long-term follow-up to assess the persistence of observed effects; (vii) variation in the effects of SUDs across different developmental stages; (viii) co-occurrence of multiple substance uses, which may lead to inconsistencies across studies.

This review has integrated genetic factors, personality traits, and epigenetic interactions mediated by the GM in the context of addiction susceptibility. To our knowledge, no previous review has addressed these domains in a similarly integrative manner, conferring a foundational character to the present review. However, precisely due to the nature of this review, it entails certain inherent limitations. First, due to the wide diversity of domains examined, a systematic methodology was not employed. Instead, a narrative approach was adopted, which allowed for broader conceptual synthesis but may be subject to selection bias and potentially limit replicability. Second, the integration of multiple, highly heterogeneous areas may have led to simplifications or omissions in the discussion of specific mechanisms, potentially limiting the specificity of the conclusions. Third, differences in study design, sample characteristics, and measurement methods across the cited studies could contribute to high variability. Finally, the rapidly evolving landscape of research may limit the generalizability of the findings over time.

## 7. Conclusions

Genetic influences on personality act primarily via regulatory variants that modulate gene expression during neurodevelopment, shaping cognitive, emotional, and behavioral traits that contribute to individual differences in vulnerability to psychiatric conditions and adaptive responses to environmental challenges. SUDs share partially overlapping genetic foundations, with specific loci, heritability estimates, and causal pathways differing across substances, reflecting both shared vulnerability and substance-specific genetic influences on susceptibility to SUDs. Heritable personality traits, particularly impulsivity, novelty-seeking, and stress responsiveness, interact to shape susceptibility to SUDs, with genetic factors modulating risk across different forms of addiction. Thus, a heritable personality profile characterized by these traits may confer heightened vulnerability to SUDs, especially under challenging or adverse environmental conditions, but not deterministically leading to addictive behaviors. In addition, environmental factors, ELS, and social influences interact with the GM to shape neurobiological and behavioral pathways that modulate addiction risk. These interactions highlight the multifactorial nature of SUDs, in which epigenetic, microbial, and psychosocial mechanisms converge to influence susceptibility, progression, and maintenance of addictive behaviors. Integrating multi-omics approaches, including metagenomics, metabolomics, and host transcriptomics, can elucidate how GM functions influence host gene expression, advancing our understanding of microbial contributions to addiction susceptibility.

## Figures and Tables

**Table 1:** SUD genes and variants, SUD molecular genetic findings, and SUD genetic epidemiology.

Features	AUD	CUD	OUD	TUD
GWAS risk-loci	*ADH1B* rs1229984 [141]	*CHRNA2* rs4732724 [120]	*BEND4* (gene-wise) [142]	*CHRNA4* rs151176846 [127]
	*ALDH2* rs671 [103]	*CSMD1* rs77378271 [136]	*CNIH3* rs10799590 [124]	*CHRNA5* rs16969968 [128]
	*DRD2* rs4936277 [106]	*EPHX2* rs4732724 [118]	*KCNG2* rs62103177 [123]	*CHRNA5-A3-B4* (multiple loci) [126]
	*GCKR* rs1260326 [143]	*FOXP2* rs7783012 [118]	*OPRM1* rs1799971 [108]	*DBH* rs13284520 [128]
	*KLB* rs13129401 [108]	*PDE4B* (gene-wise) [102]	*RGMA* rs12442183 [122]	*DNMT3B* rs910083 [129]
	*SLC39A8* rs13107325 [143]			
	Drinks per week (rg = +0.77) [84]	AUD (rg = +0.55) [118]	ADHD (rg = +0.36) [108]	Alcohol dependence (rg = +0.56) [128]
	Ever smoked regularly (rg = +0.55) [84]	Educational attainment (rg = −0.39) [118]	Alcohol dependence (rg = +0.73) [108]	Cigarettes per day (rg = +0.95) [128]
Genetic correlations with	Lifetime cannabis use (rg = +0.39) [84]	Lifetime cannabis use (rg = +0.50) [118]	Drinks per week (rg = +0.38) [108]	MDD (rg = +0.38) [128]
	MDD (rg = +0.39) [84]	Schizophrenia (rg = +0.31) [118]	Ever smoked regularly (rg = +0.51) [108]	Schizophrenia (rg = +0.16) [128]
	Risk-taking (rg = +0.30) [84]	Smoking initiation (rg = +0.66) [118]	MDD (rg = +0.35) [108]	Smoking initiation (rg = +0.40) [128]
SUD genetic epidemiology	AUD heritability (h^2^) = 0.50 [131]	CUD heritability (h^2^) = 0.48–0.51 [136]		
	Alcohol use initiation (h^2^ = 0.37) [132]	Cannabis use/initiation (h^2^ = 0.30–0.50) [137]	OUD heritability (h^2^) = 0.34–0.50 [139,140]	TUD heritability (h^2^) = 0.30–0.70 [134,135]
	Alcohol use frequency (h^2^ = 0.37–0.50) [133]			

*ADH1B*: alcohol dehydrogenase 1B gene; *ALDH2*: aldehyde dehydrogenase 2 gene; *BEND4*: BEN domain containing 4 gene; *CHRNA2*: cholinergic receptor nicotinic alpha 2 subunit gene; *CHRNA4*: cholinergic receptor nicotinic alpha 4 subunit gene; *CHRNA5*: cholinergic receptor nicotinic alpha 5 subunit gene; *CNIH3*: Cornichon family AMPA receptor auxiliary protein 3 gene; *CSMD1*: CUB and Sushi multiple domains 1 gene; *DBH*: dopamine beta-hydroxylase gene; *DNMT3B*: DNA methyltransferase 3 beta gene; *DRD2*: dopaminergic receptor D2 gene; *EPHX2*: epoxide hydrolase gene; *FOXP2*: forkhead box P2 gene; *GCKR*: glucokinase regulator gene; *KCNG2*: potassium voltage-gated channel modifier subfamily G member 2 gene; *KLB*: Klotho beta gene; *OPRM1*: opioid receptor mu 1 gene; *PDE4B*: phosphodiesterase 4B gene; *RGMA*: repulsive guidance molecule A gene; *SLC39A8*: solute carrier gene family 39, member 8; rg: genetic correlation. MDD: major depression disorder; ADHD: attention deficit hyperactivity disorder.

**Table 2:** Microbial associations with personality traits in childhood and adulthood.

Traits	Childhood	Association	Adults	Association	References
	Negative	Positive	Negative	Positive	
Surgency, Extraversion and Openness	*Bacteroides*	*Enterobacteriaceae* *Rikenellaceae* *Ruminococcacea* *Bifidobacterium* *Dialister* *Parabacteroides* *Streptococcus*	*Fusobacterium*	NE	[261,262,263]
Emotional reactivity	NE	*Bifidobacterium* *Erwinia* *Rothia* *Serratia* *Streptococcus*	NE	NE	[261,264,265]
Fear response/ Threat acquisition	*Bacteroides* *Clostridium*	*Lachnospiraceae* *Rikenellaceae* *Atopobium* *Bifidobacterium* *Bilophila* *Dialister* *Lactobacillus* *Parabacteroides* *Peptoniphilus* *Roseburia* *Veillonella*	NE	*Agathobacter* *Alistipes* *Bacteroides* *Butyricicoccus* *Faecalibacterium* *Phocea* *Tyzzerella* *Veillonella*	[261,262,264,265,266,267,268]
Neuroticism/ Depression	NE	*Akkermansia*	*Corynebacterium* *Megamonas* *Odoribacter* *Streptococcus*	Members of the phylum Pseudomonadota *Bifidobacterium* *Desulfovibrio* *Haemophilus*	[257,258,259,263,265,269,270]
Conscientiousness/ Attentional focusing	*Alistipes*	NE	Members of the phylum Pseudomonadota *Alistipes* *Megamonas* *Sudoligranulum*	*Lachnospiraceae* *Lachnospira*	[257,259,263,271]
Sociability/ Cuddliness/ Soothability	*Hungatella*	*Akkermansia* *Bifidobacterium*	*Desulfovibrio* *Sutterella*	*Akkermansia* *Lactococcus* *Oscillospira*	[258,271,272,273]
Happiness	NE	NE	NE	*Bifidobacterium* *Clostridium*	[269]

NE: not established.

## Data Availability

No new data were created or analyzed in this study. Data sharing is not applicable to this article.

## Abbreviations

The following abbreviations are used in this manuscript:

ACEs	Adverse childhood experiences
ADHD	Attention-deficit/hyperactivity disorder
ASD	Autism spectrum disorder
AUD	Alcohol use disorder
AUDIT	Alcohol use disorder identification test
BAs	Bile acids
BBB	Blood–brain barrier
BD	Bipolar disorder
BMI	Body mass index
ChIP	Chromatin immunoprecipitation
CNR1	Cannabinoid receptor 1
CUD	Cannabis use disorder
DNAm	DNA methylation
DRD2	Dopamine D2 receptor
ELS	Early-life stress
eQTLs	Expression quantitative trait loci
EWAS	Epigenome-wide association study
FMT	Fecal microbiota transplantation
GBA	Gut–brain axis
GD	Gambling disorder
GM	Gut microbiome
GWAS	Genome-wide association studies
HPA	Hypothalamic–pituitary–adrenal
IPTs	Impulsive personality traits
KYNA	Kynurenic acid
MDD	Major depressive disorder
MPRAs	Massively parallel reporter assays
MR	Mendelian randomization
NAc	Nucleus accumbens
NEO-FFI	NEO Five-Factor Inventory
ND	Nicotine dependence
NGS	Next-generation sequencing
NS	Novelty seeking
OFC	Orbitofrontal cortex
OUD	Opioid use disorder
PAU	Problematic alcohol use
PGC	Psychiatric Genomics Consortium
PGS	Polygenic scores
PTSD	Post-traumatic stress disorder
RCT	Randomized controlled trial
RD	Reward dependence
rg	Genetic correlation
rsFC	Resting-state functional connectivity
SCFAs	Short-chain fatty acids
SES	Socioeconomic status
SNP	Single-nucleotide polymorphism
STAI	State-Trait Anxiety Inventory
SUD	Substance use disorders
TUD	Tobacco use disorder
VTA	Ventral tegmental area

## References

[1] Alavi S.S., Ferdosi M., Jannatifard F., Eslami M., Alaghemandan H., Setare M. (2012). Behavioral Addiction versus Substance Addiction: Correspondence of Psychiatric and Psychological Views. Int. J. Prevent. Med..

[2] Zilberman N., Yadid G., Efrati Y., Neumark Y., Rassovsky Y. (2018). Personality profiles of substance and behavioral addictions. Addict. Behav..

[3] American Psychiatric Association, DSM-5 Task Force (2013). Diagnostic and Statistical Manual of Mental Disorders: DSM-5™.

[4] Hasin D.S., O’Brien C.P., Auriacombe M., Borges G., Bucholz K., Budney A., Compton W.M., Crowley T., Ling W., Petry N.M. (2013). DSM-5 criteria for substance use disorders: Recommendations and rationale. Am. J. Psychiatry.

[5] Compton W.M., Dawson D.A., Goldstein R.B., Grant B.F. (2013). Crosswalk between DSM-IV dependence and DSM-5 substance use disorders for opioids, cannabis, cocaine and alcohol. Drug Alcohol Depend..

[6] Alcaro A., Brennan A., Conversi D. (2021). The SEEKING Drive and Its Fixation: A Neuro-Psycho-Evolutionary Approach to the Pathology of Addiction. Front. Hum. Neurosci..

[7] Popescu A., Marian M., Drăgoi A.M., Costea R.V. (2021). Understanding the genetics and neurobiological pathways behind addiction (Review). Exp. Ther. Med..

[8] Morrow J.D., Flagel S.B. (2016). Neuroscience of resilience and vulnerability for addiction medicine: From genes to behavior. Prog. Brain Res..

[9] Belfiore C.I., Galofaro V., Cotroneo D., Lopis A., Tringali I., Denaro V., Casu M. (2024). A Multi-Level Analysis of Biological, Social, and Psychological Determinants of Substance Use Disorder and Co-Occurring Mental Health Outcomes. Psychoactives.

[10] Magidson J.F., Liu S.M., Lejuez C.W., Blanco C. (2012). Comparison of the Course of Substance Use Disorders among Individuals With and Without Generalized Anxiety Disorder in a Nationally Representative Sample. J. Psychiatr. Res..

[11] Forster G.L., Anderson E.M., Scholl J.L., Lukkes J.L., Watt M.J. (2018). Negative consequences of early-life adversity on substance use as mediated by corticotropin-releasing factor modulation of serotonin activity. Neurobiol. Stress.

[12] Mitchell M.R., Potenza M.N. (2014). Addictions and Personality Traits: Impulsivity and Related Constructs. Curr. Behav. Neurosci. Rep..

[13] He J., Yan X., Wang R., Zhao J., Liu J., Zhou C., Zeng Y. (2022). Does Childhood Adversity Lead to Drug Addiction in Adulthood? A Study of Serial Mediators Based on Resilience and Depression. Front. Psychiatry.

[14] Liu S.X., Harris A.C., Gewirtz J.C. (2024). How life events may confer vulnerability to addiction: The role of epigenetics. Front. Mol. Neurosci..

[15] Morales A.M., Jones S.A., Kliamovich D., Harman G., Nagel B.J. (2020). Identifying Early Risk Factors for Addiction Later in Life: A Review of Prospective Longitudinal Studies. Curr. Addict. Rep..

[16] Saladino V., Mosca O., Petruccelli F., Hoelzlhammer L., Lauriola M., Verrastro V., Cabras C. (2021). The Vicious Cycle: Problematic Family Relations, Substance Abuse, and Crime in Adolescence: A Narrative Review. Front. Psychol..

[17] Tzouvara V., Kupdere P., Wilson K., Matthews L., Simpson A., Foye U. (2023). Adverse childhood experiences, mental health, and social functioning: A scoping review of the literature. Child Abus. Negl..

[18] Borrego-Ruiz A. (2024). Motivación intrínseca y consumo de drogas: Una revisión de estudios sobre los motivos de curiosidad y de expansión [Intrinsic motivation and drug consumption: A review of studies on curiosity and expansion motives]. Salud Y Drog. /Health Addict..

[19] Belcher A.M., Volkow N.D., Moeller F.G., Ferré S. (2014). Personality traits and vulnerability or resilience to substance use disorders. Trends Cogn. Sci..

[20] Kandler C., Richter J., Zapko-Willmes A., Zeigler-Hill V., Shackelford T. (2017). Genetic Basis of Traits. Encyclopedia of Personality and Individual Differences.

[21] Soto C.J., Bornstein M.H., Arterberry M.E., Fingerman K.L., Lansford J.E. (2018). Big Five personality traits. The SAGE Encyclopedia of Lifespan Human Development.

[22] Widiger T.A., Crego C. (2019). The Five Factor Model of personality structure: An update. World Psychiatry.

[23] Amodeo M. (2015). The Addictive Personality. Subst. Use Misuse.

[24] Lafta M.S., Mwinyi J., Affatato O., Rukh G., Dang J., Andersson G., Schiöth H.B. (2024). Exploring sex differences: Insights into gene expression, neuroanatomy, neurochemistry, cognition, and pathology. Front. Neurosci..

[25] Chapman B.P., Duberstein P.R., Sorensen S., Lyness J.M. (2007). Gender Differences in Five Factor Model Personality Traits in an Elderly Cohort. Personal. Individ. Differ..

[26] Olino T.M., Durbin C.E., Klein D.N., Hayden E.P., Dyson M.W. (2013). Gender differences in young children’s temperament traits: Comparisons across observational and parent-report methods. J. Pers..

[27] Costa P.T., Terracciano A., McCrae R.R. (2001). Gender Differences in Personality Traits across Cultures: Robust and Surprising Findings. J. Personal. Soc. Psychol..

[28] Casale S., Carducci B.J., Nave C.S., Di Fabio A., Saklofske D.H., Con Stough C. (2020). Gender Differences in Self-esteem and Self-confidence. Personality Processes and Individuals Differences of The Wiley Encyclopedia of Personality and Individual Differences.

[29] Tschan T., Peter-Ruf C., Schmid M., In-Albon T. (2017). Temperament and character traits in female adolescents with nonsuicidal self-injury disorder with and without comorbid borderline personality disorder. Child Adolesc. Psychiatry Ment. Health.

[30] Ducci F., Goldman D. (2012). The genetic basis of addictive disorders. Psychiatr. Clin. N. Am..

[31] Volkow N.D., Muenke M. (2012). The genetics of addiction. Hum. Genet..

[32] Vink J.M. (2016). Genetics of Addiction: Future Focus on Gene × Environment Interaction?. J. Stud. Alcohol Drugs.

[33] Armani S., Geier A., Forst T., Merle U., Alpers D.H., Lunnon M.W. (2024). Effect of changes in metabolic enzymes and transporters on drug metabolism in the context of liver disease: Impact on pharmacokinetics and drug-drug interactions. Br. J. Clin. Pharmacol..

[34] Borrego-Ruiz A., Borrego J.J. (2025). Pharmacogenomic and Pharmacomicrobiomic Aspects of Drugs of Abuse. Genes.

[35] Casiano N. (2025). Epigenetic Implications of Substance Abuse in Latino Populations: A Review of Substance-Specific and Intergenerational Outcomes. J. Soc. Work Welf. Policy.

[36] Virolainen S.J., VonHandorf A., Viel K.C.M.F., Weirauch M.T., Kottyan L.C. (2023). Gene-environment interactions and their impact on human health. Genes Immun..

[37] Borrego-Ruiz A., Borrego J.J. (2025). The Bidirectional Interplay Between Substances of Abuse and Gut Microbiome Homeostasis. Life.

[38] García-Cabrerizo R., Cryan J.F. (2024). A gut (microbiome) feeling about addiction: Interactions with stress and social systems. Neurobiol. Stress.

[39] Russell J.T., Zhou Y., Weinstock G.M., Bubier J.A. (2021). The Gut Microbiome and Substance Use Disorder. Front. Neurosci..

[40] Kazemian N., Pakpour S. (2024). Understanding the impact of the gut microbiome on opioid use disorder: Pathways, mechanisms, and treatment insights. Microb. Biotechnol..

[41] Chen G., Shi F., Yin W., Guo Y., Liu A., Shuai J., Sun J. (2022). Gut microbiota dysbiosis: The potential mechanisms by which alcohol disrupts gut and brain functions. Front. Microbiol..

[42] Jadhav K.S., Peterson V.L., Halfon O., Ahern G., Fouhy F., Stanton C., Dinan T.G., Cryan J.F., Boutrel B. (2018). Gut microbiome correlates with altered striatal dopamine receptor expression in a model of compulsive alcohol seeking. Neuropharmacology.

[43] Xiao H.W., Ge C., Feng G.X., Li Y., Luo D., Dong J.L., Li H., Wang H., Cui M., Fan S.J. (2018). Gut microbiota modulates alcohol withdrawal-induced anxiety in mice. Toxicol. Lett..

[44] Gagliano S.A. (2017). It’s all in the brain: A review of available functional genomic annotations. Biol. Psychiatry.

[45] Boutwell B.B., White M.A. (2019). Gene regulation and the architecture of complex human traits in the genomics era. Curr. Opin. Psychol..

[46] de la Torre-Ubieta L., Stein J.L., Won H., Opland C.K., Liang D., Lu D., Geschwind D.H. (2018). The dynamic landscape of open chromatin during human cortical neurogenesis. Cell.

[47] Skene N.G., Bryois J., Bakken T.E., Breen G., Crowley J.J., Gaspar H.A., Giusti-Rodriguez P., Hodge R.D., Miller J.A., Muñoz-Manchado A.B. (2018). Genetic identification of brain cell types underlying schizophrenia. Nat. Genet..

[48] Williams S.M., An J.Y., Edson J., Watts M., Murigneux V., Whitehouse A.J.O., Jackson C.J., Bellgrove M.A., Cristino A.S., Claudianos C. (2019). An integrative analysis of non-coding regulatory DNA variations associated with autism spectrum disorder. Mol. Psychiatry.

[49] Deshpande D., Chhugani K., Chang Y., Karlsberg A., Loeffler C., Zhang J., Muszyńska A., Munteanu V., Yang H., Rotman J. (2023). RNA-seq data science: From raw data to effective interpretation. Front. Genet..

[50] Liu Y., Fu L., Kaufmann K., Chen D., Chen M. (2019). A practical guide for DNase-seq data analysis: From data management to common applications. Brief. Bioinform..

[51] Buenrostro J.D., Wu B., Chang H.Y., Greenleaf W.J. (2015). ATAC-seq: A Method for Assaying Chromatin Accessibility Genome-Wide. Curr. Protoc. Mol. Biol..

[52] Hino S., Sato T., Nakao M. (2023). Chromatin Immunoprecipitation Sequencing (ChIP-seq) for Detecting Histone Modifications and Modifiers. Methods Mol. Biol..

[53] Kundaje A., Meuleman W., Ernst J., Bilenky M., Yen A., Heravi-Moussavi A., Kheradpour P., Zhang Z., Wang J., Roadmap Epigenomics Consortium (2015). Integrative analysis of 111 reference human epigenomes. Nature.

[54] Higgins G.A., Allyn-Feuer A., Athey B.D. (2015). Epigenomic mapping and effect sizes of noncoding variants associated with psychotropic drug response. Pharmacogenomics.

[55] Orozco G., Schoenfelder S., Walker N., Eyre S., Fraser P. (2022). 3D genome organization links non-coding disease-associated variants to genes. Front. Cell Dev. Biol..

[56] White M.A. (2015). Understanding how cis-regulatory function is encoded in DNA sequence using massively parallel reporter assays and designed sequences. Genomics.

[57] Borrego-Ruiz A. (2024). A holistic review of fentanyl use and its impact on public health. Curr. Addict. Res..

[58] Nguyen P.L.L., Syed M., McGue M. (2021). Behavior genetics research on personality: Moving beyond traits to examine characteristic adaptations. Soc. Pers. Psychol. Compass.

[59] Rima D., Sholpan M., Gulzagira A., Meruert B., Beaver K.M. (2020). Exploring the potential association between gang membership and health outcomes in a longitudinal sample of youth and young adults. J. Crim. Justice.

[60] Yablonska T., Yuanjie L. (2023). Psychological characteristics of young men with Internet addiction: A cross-cultural study. Pedagog. Psychol..

[61] Spytska L. (2025). Psychogenetic Features of the Influence of Genes on Human Life. Arch. Psychiatry Res..

[62] Zwir I., Del-Val C., Arnedo J., Pulkki-Råback L., Konte B., Yang S.S., Romero-Zaliz R., Hintsanen M., Cloninger K.M., Garcia D. (2021). Three genetic-environmental networks for human personality. Mol. Psychiatry.

[63] Sallis H., Davey Smith G., Munafò M.R. (2018). Genetics of biologically based psychological differences. Philos. Trans. R. Soc. Lond. B Biol. Sci..

[64] Munafò M.R., Flint J. (2014). Common or rare variants for complex traits?. Biol. Psychiatry.

[65] Xiao X., Chang H., Li M. (2017). Molecular mechanisms underlying noncoding risk variations in psychiatric genetic studies. Mol. Psychiatry.

[66] Arner E., Daub C.O., Vitting-Seerup K., Andersson R., Lilje B., Drabløs F., Lennartsson A., Rönnerblad M., Hrydziuszko O., Vitezic M. (2015). Transcribed enhancers lead waves of coordinated transcription in transitioning mammalian cells. Science.

[67] Doan R.N., Bae B.I., Cubelos B., Chang C., Hossain A.A., Al-Saad S., Mukaddes N.M., Oner O., Al-Saffar M., Balkhy S. (2016). Mutations in human accelerated regions disrupt cognition and social behavior. Cell.

[68] Gamazon E.R., Badner J.A., Cheng L., Zhang C., Zhang D., Cox N.J., Gershon E.S., Kelsoe J.R., Greenwood T.A., Nievergelt C.M. (2013). Enrichment of cis-regulatory gene expression SNPs and methylation quantitative trait loci among bipolar disorder susceptibility variants. Mol. Psychiatry.

[69] Short P.J., McRae J.F., Gallone G., Sifrim A., Won H., Geschwind D.H., Wright C.F., Firth H.V., FitzPatrick D.R., Barrett J.C. (2018). De novo mutations in regulatory elements in neurodevelopmental disorders. Nature.

[70] Zehra R., Abbasi A.A. (2018). Homo sapiens-specific binding site variants within brain exclusive enhancers are subject to accelerated divergence across human population. Genome Biol. Evol..

[71] Del Val C., Díaz de la Guardia-Bolívar E., Zwir I., Mishra P.P., Mesa A., Salas R., Poblete G.F., de Erausquin G., Raitoharju E., Kähönen M. (2024). Gene expression networks regulated by human personality. Mol. Psychiatry.

[72] Zwir I., Arnedo J., Mesa A., Del Val C., de Erausquin G.A., Cloninger C.R. (2023). Temperament & Character account for brain functional connectivity at rest: A diathesis-stress model of functional dysregulation in psychosis. Mol. Psychiatry.

[73] Liu M., Jiang Y., Wedow R., Li Y., Brazel D.M., Chen F., Datta G., Davila-Velderrain J., McGuire D., Tian C. (2019). Association studies of up to 1.2 million individuals yield new insights into the genetic etiology of tobacco and alcohol use. Nat. Genet..

[74] Stringer S., Minică C.C., Verweij K.J.H., Mbarek H., Bernard M., Derringer J., van Eijk K.R., Isen J.D., Loukola A., Maciejewski D.F. (2016). Genome-wide association study of lifetime cannabis use based on a large metaanalytic sample of 32 330 subjects from the International Cannabis Consortium. Transl. Psychiatry.

[75] Saunders G.R.B., Wang X., Chen F., Jang S.K., Liu M., Wang C., Gao S., Jiang Y., Khunsriraksakul C., Otto J.M. (2022). Genetic diversity fuels gene discovery for tobacco and alcohol use. Nature.

[76] Pasman J.A., Verweij K.J.H., Gerring Z., Stringer S., Sanchez-Roige S., Treur J.L., Abdellaoui A., Nivard M.G., Baselmans B.M.L., Ong J.S. (2018). GWAS of lifetime cannabis use reveals new risk loci, genetic overlap with psychiatric traits, and a causal effect of schizophrenia liability. Nat. Neurosci..

[77] Minică C.C., Verweij K.J.H., van der Most P.J., Mbarek H., Bernard M., van Eijk K.R., Lind P.A., Liu M.Z., Maciejewski D.F., Palviainen T. (2018). Genome-wide association meta-analysis of age at first cannabis use. Addiction.

[78] Gerring Z.F., Thorp J.G., Treur J.L., Verweij K.J.H., Derks E.M. (2024). The genetic landscape of substance use disorders. Mol. Psychiatry.

[79] Sanchez-Roige S., Palmer A.A., Fontanillas P., Elson S.L., Adams M.J., Howard D.M., Edenberg H.J., Davies G., Crist R.C., 23andMe Research Team, the Substance Use Disorder Working Group of the Psychiatric Genomics Consortium (2019). Genome-Wide Association Study Meta-Analysis of the Alcohol Use Disorders Identification Test (AUDIT) in Two Population-Based Cohorts. Am. J. Psychiatry.

[80] Toikumo S., Jennings M.V., Pham B.K., Lee H., Mallard T.T., Bianchi S.B., Meredith J.J., Vilar-Ribó L., Xu H., Hatoum A.S. (2024). Multi-ancestry meta-analysis of tobacco use disorder identifies 461 potential risk genes and reveals associations with multiple health outcomes. Nat. Hum. Behav..

[81] Kember R.L., Davis C.N., Feuer K.L., Kranzler H.R. (2024). Considerations for the application of polygenic scores to clinical care of individuals with substance use disorders. J. Clin. Investig..

[82] Ma Y., Zhou X. (2021). Genetic prediction of complex traits with polygenic scores: A statistical review. Trends Genet..

[83] Murray G.K., Lin T., Austin J., McGrath J.J., Hickie I.B., Wray N.R. (2021). Could polygenic risk scores be useful in psychiatry?: A review. JAMA Psychiatry.

[84] Zhou H., Sealock J.M., Sanchez-Roige S., Clarke T.K., Levey D.F., Cheng Z., Li B., Polimanti R., Kember R.L., Smith R.V. (2020). Genome-wide meta-analysis of problematic alcohol use in 435,563 individuals yields insights into biology and relationships with other traits. Nat. Neurosci..

[85] Deak J.D., Zhou H., Galimberti M., Levey D.F., Wendt F.R., Sanchez-Roige S., Hatoum A.S., Johnson E.C., Nunez Y.Z., Demontis D. (2022). Genome-wide association study in individuals of European and African ancestry and multi-trait analysis of opioid use disorder identifies 19 independent genome-wide significant risk loci. Mol. Psychiatry.

[86] Shi Y., Mota N.R., Franke B., Sprooten E. (2025). Genetic liability to major psychiatric disorders contributes to multi-faceted quality of life outcomes in children and adults. Transl. Psychiatry.

[87] Johnson E.C., Sanchez-Roige S., Acion L., Adams M.J., Bucholz K.K., Chan G., Chao M.J., Chorlian D.B., Dick D.M., Edenberg H.J. (2021). Polygenic contributions to alcohol use and alcohol use disorders across population-based and clinically ascertained samples. Psychol. Med..

[88] Wang F.L., Hicks B.M., Zhou H., Kranzler H.R., Gelernter J., Zucker R.A. (2023). Polygenic risk score for problematic alcohol use predicts heavy drinking and alcohol use disorder symptoms in young adulthood after accounting for adolescent alcohol use and parental alcohol use disorder. Drug Alcohol Depend..

[89] Yeung E.W., Spychala K.M., Miller A.P., Otto J.M., Deak J.D., Kim H., Gilder D.A., Ehlers C.L., Wilhelmsen K.C., Gizer I.R. (2022). Effects of genetic risk for alcohol dependence and onset of regular drinking on the progression to alcohol dependence: A polygenic risk score approach. Drug Alcohol Depend..

[90] Li J.J., Cho S.B., Salvatore J.E., Edenberg H.J., Agrawal A., Chorlian D.B., Porjesz B., Hesselbrock V., Dick D.M., COGA Investigators (2017). The impact of peer substance use and polygenic risk on trajectories of heavy episodic drinking across adolescence and emerging adulthood. Alcohol Clin. Exp. Res..

[91] Su J., Trevino A., Jamil B., Aliev F. (2022). Genetic risk of AUDs and childhood impulsivity: Examining the role of parenting and family environment. Dev. Psychopathol..

[92] Kranzler H.R., Feinn R., Xu H., Ho B.L., Saini D., Nicastro O.R., Jacoby A., Toikumo S., Gelernter J., Hartwell E.E. (2023). Does polygenic risk for substance-related traits predict ages of onset and progression of symptoms?. Addiction.

[93] Kember R.L., Hartwell E.E., Xu H., Rotenberg J., Almasy L., Zhou H., Gelernter J., Kranzler H.R. (2023). Phenome-wide association analysis of substance use disorders in a deeply phenotyped sample. Biol. Psychiatry.

[94] Vilar-Ribó L., Sánchez-Mora C., Rovira P., Richarte V., Corrales M., Fadeuilhe C., Arribas L., Casas M., Ramos-Quiroga J.A., Ribasés M. (2021). Genetic overlap and causality between substance use disorder and attentiondeficit and hyperactivity disorder. Am. J. Med. Genet. B Neuropsychiatr. Genet..

[95] Su J., Trevino A.D., Kuo S.I.C., Aliev F., Williams C.D., Guy M.C., Dick D.M., Spit for Science Working Group (2022). Racial discrimination and alcohol problems: Examining interactions with genetic risk and impulsivity among African American young adults. J. Youth Adolesc..

[96] Cusack S.E., Aliev F., Bustamante D., Dick D.M., Amstadter A.B. (2023). A statistical genetic investigation of psychiatric resilience. Eur. J. Psychotraumatol..

[97] Meyers J.L., Chorlian D.B., Johnson E.C., Pandey A.K., Kamarajan C., Salvatore J.E., Aliev F., Subbie-Saenz de Viteri S., Zhang J., Chao M. (2019). Association of Polygenic Liability for Alcohol Dependence and EEG Connectivity in Adolescence and Young Adulthood. Brain Sci..

[98] Segura A.G., Mané A., Prohens L., Rodriguez N., Mezquida G., Cuesta M.J., Vieta E., Amoretti S., Lobo A., González-Pinto A. (2023). Exploration of cannabis use and polygenic risk scores on the psychotic symptom progression of a FEP cohort. Psychiatry Res..

[99] Cheng W., Parker N., Karadag N., Koch E., Hindley G., Icick R., Shadrin A., O’Connell K.S., Bjella T., Bahrami S. (2023). The relationship between cannabis use, schizophrenia, and bipolar disorder: A genetically informed study. Lancet Psychiatry.

[100] Paul S.E., Hatoum A.S., Barch D.M., Thompson W.K., Agrawal A., Bogdan R., Johnson E.C. (2022). Associations between cognition and polygenic liability to substance involvement in middle childhood: Results from the ABCD study. Drug Alcohol Depend..

[101] Hartwell E.E., Merikangas A.K., Verma S.S., Ritchie M.D., Kranzler H.R., Kember R.L., Regeneron Genetics Center (2022). Genetic liability for substance use associated with medical comorbidities in electronic health records of African- and European-ancestry individuals. Addict. Biol..

[102] Hatoum A.S., Colbert S.M.C., Johnson E.C., Huggett S.B., Deak J.D., Pathak G., Jennings M.V., Paul S.E., Karcher N.R., Hansen I. (2023). Multivariate genome-wide association meta-analysis of over 1 million subjects identifies loci underlying multiple substance use disorders. Nat. Ment. Health.

[103] Edenberg H.J., Mcclintick J.N. (2018). Alcohol dehydrogenases, aldehyde dehydrogenases, and alcohol use disorders: A critical review. Alcohol. Clin. Exp. Res..

[104] Deak J.D., Miller A.P., Gizer I.R. (2019). Genetics of alcohol use disorder: A review. Curr. Opin. Psychol..

[105] Sanchez-Roige S., Palmer A.A., Clarke T.K. (2020). Recent efforts to dissect the genetic basis of alcohol use and abuse. Biol. Psychiatry.

[106] Kranzler H.R., Zhou H., Kember R.L., Vickers Smith R., Justice A.C., Damrauer S., Tsao P.S., Klarin D., Baras A., Reid J. (2019). Genome-wide association study of alcohol consumption and use disorder in 274424 individuals from multiple populations. Nat. Commun..

[107] Walters R.K., Polimanti R., Johnson E.C., McClintick J.N., Adams M.J., Adkins A.E., Aliev F., Bacanu S.A., Batzler A., Bertelsen S. (2018). Transancestral GWAS of alcohol dependence reveals common genetic underpinnings with psychiatric disorders. Nat. Neurosci..

[108] Zhou H., Rentsch C.T., Cheng Z., Kember R.L., Nunez Y.Z., Sherva R.M., Tate J.P., Dao C., Xu K., Polimanti R. (2020). Association of OPRM1 functional coding variant with opioid use disorder. JAMA Psychiatry.

[109] Clarke T.K., Adams M.J., Davies G., Howard D.M., Hall L.S., Padmanabhan S., Murray A.D., Smith B.H., Campbell A., Hayward C. (2017). Genome-wide association study of alcohol consumption and genetic overlap with other health-related traits in UK Biobank (N = 112 117). Mol. Psychiatry.

[110] Gelernter J., Sun N., Polimanti R., Pietrzak R.H., Levey D.F., Lu Q., Hu Y., Li B., Radhakrishnan K., Aslan M. (2019). Genome-wide association study of maximum habitual alcohol intake in >140000 US European and African American veterans yields novel risk loci. Biol. Psychiatry.

[111] Sanchez-Roige S., Fontanillas P., Elson S.L., Gray J.C., de Wit H., MacKillop J., Palmer A.A. (2019). Genome-Wide Association Studies of Impulsive Personality Traits (BIS-11 and UPPS-P) and Drug Experimentation in up to 22,861 Adult Research Participants Identify Loci in the *CACNA1I* and *CADM2* genes. J. Neurosci..

[112] Gelernter J., Zhou H., Nuñez Y.Z., Mutirangura A., Malison R.T., Kalayasiri R. (2018). Genomewide Association Study of Alcohol Dependence and Related Traits in a Thai Population. Alcohol. Clin. Exp. Res..

[113] Quillen E.E., Chen X.D., Almasy L., Yang F., He H., Li X., Wang X.Y., Liu T.Q., Hao W., Deng H.W. (2014). ALDH2 is associated to alcohol dependence and is the major genetic determinant of ‘daily maximum drinks’ in a GWAS study of an isolated rural Chinese sample. Am. J. Med. Genet. B Neuropsychiatr. Genet..

[114] Jorgenson E., Thai K.K., Hoffmann T.J., Sakoda L.C., Kvale M.N., Banda Y., Schaefer C., Risch N., Mertens J., Weisner C. (2017). Genetic contributors to variation in alcohol consumption vary by race/ethnicity in a large multi-ethnic genome-wide association study. Mol. Psychiatry.

[115] Mallard T.T., Savage J.E., Johnson E.C., Huang Y., Edwards A.C., Hottenga J.J., Grotzinger A.D., Gustavson D.E., Jennings M.V., Anokhin A. (2022). Item-Level Genome-Wide Association Study of the Alcohol Use Disorders Identification Test in Three Population-Based Cohorts. Am. J. Psychiatry.

[116] Marees A.T., Smit D.J.A., Ong J.S., MacGregor S., An J., Denys D., Vorspan F., van den Brink W., Derks E.M. (2020). Potential influence of socioeconomic status on genetic correlations between alcohol consumption measures and mental health. Psychol. Med..

[117] Deak J.D., Johnson E.C. (2021). Genetics of substance use disorders: A review. Psychol. Med..

[118] Johnson E.C., Demontis D., Thorgeirsson T.E., Walters R.K., Polimanti R., Hatoum A.S., Sanchez-Roige S., Paul S.E., Wendt F.R., Clarke T.K. (2020). A large-scale genome-wide association study meta-analysis of cannabis use disorder. Lancet Psychiatry.

[119] den Hoed J., Devaraju K., Fisher S.E. (2021). Molecular networks of the FOXP2 transcription factor in the brain. EMBO Rep..

[120] Demontis D., Rajagopal V.M., Thorgeirsson T.E., Als T.D., Grove J., Leppälä K., Gudbjartsson D.F., Pallesen J., Hjorthøj C., Reginsson G.W. (2019). Genome-wide association study implicates CHRNA2 in cannabis use disorder. Nat. Neurosci..

[121] Sherva R., Wang Q., Kranzler H., Zhao H., Koesterer R., Herman A., Farrer L.A., Gelernter J. (2016). Genome-Wide Association Study of Cannabis Dependence Severity, Novel Risk Variants, and Shared Genetic Risks. JAMA Psychiatry.

[122] Cheng Z., Zhou H., Sherva R., Farrer L.A., Kranzler H.R., Gelernter J. (2018). Genome-wide association study identifies a regulatory variant of *RGMA* associated with opioid dependence in European Americans. Biol. Psychiatry.

[123] Gelernter J., Kranzler H.R., Sherva R., Koesterer R., Almasy L., Zhao H., Farrer L.A. (2014). Genome-wide association study of opioid dependence: Multiple associations mapped to calcium and potassium pathways. Biol. Psychiatry.

[124] Nelson E.C., Agrawal A., Heath A.C., Bogdan R., Sherva R., Zhang B., Al-Hasani R., Bruchas M.R., Chou Y.L., Demers C.H. (2016). Evidence of CNIH3 involvement in opioid dependence. Mol. Psychiatry.

[125] Polimanti R., Walters R.K., Johnson E.C., McClintick J.N., Adkins A.E., Adkins D.E., Bacanu S.A., Bierut L.J., Bigdeli T.B., Brown S. (2020). Leveraging genome-wide data to investigate differences between opioid use vs. opioid dependence in 41176 individuals from the Psychiatric Genomics Consortium. Mol. Psychiatry.

[126] Hancock D.B., Markunas C.A., Bierut L.J., Johnson E.O. (2018). Human genetics of addiction: New insights and future directions. Curr. Psychiatry Rep..

[127] Hancock D.B., Reginsson G.W., Gaddis N.C., Chen X., Saccone N.L., Lutz S.M., Qaiser B., Sherva R., Steinberg S., Zink F. (2015). Genome-wide meta-analysis reveals common splice site acceptor variant in CHRNA4 associated with nicotine dependence. Transl. Psychiatry.

[128] Quach B.C., Bray M.J., Gaddis N.C., Liu M., Palviainen T., Minica C.C., Zellers S., Sherva R., Aliev F., Nothnagel M. (2020). Expanding the genetic architecture of nicotine dependence and its shared genetics with multiple traits. Nat. Commun..

[129] Hancock D.B., Guo Y., Reginsson G.W., Gaddis N.C., Lutz S.M., Sherva R., Loukola A., Minica C.C., Markunas C.A., Han Y. (2018). Genome-wide association study across European and African American ancestries identifies a SNP in *DNMT3B* contributing to nicotine dependence. Mol. Psychiatry.

[130] Prom-Wormley E.C., Ebejer J., Dick D.M., Bowers M.S. (2017). The genetic epidemiology of substance use disorder: A review. Drug Alcohol Depend..

[131] Verhulst B., Neale M.C., Kendler K.S. (2015). The heritability of alcohol use disorders: A meta-analysis of twin and adoption studies. Psychol. Med..

[132] Ystrom E., Kendler K.S., Reichborn-Kjennerud T. (2014). Early age of alcohol initiation is not the cause of alcohol use disorders in adulthood, but is a major indicator of genetic risk. A population-based twin study. Addiction.

[133] Hildebrand Karlén M., Lindqvist Bagge A.S., Berggren U., Fahlke C., Andiné P., Doering S., Lundström S. (2023). Prevalence and heritability of alcohol use disorders in 18-year old Swedish twins. Nord. Alkohol Nark..

[134] Agrawal A., Verweij K.J., Gillespie N.A., Heath A.C., Lessov-Schlaggar C.N., Martin N.G., Nelson E.C., Slutske W.S., Whitfield J.B., Lynskey M.T. (2012). The genetics of addiction—A translational perspective. Transl. Psychiatry.

[135] Goldberg L.R., Gould T.J. (2022). Genetic influences impacting nicotine use and abuse during adolescence: Insights from human and rodent studies. Brain Res. Bull..

[136] Verweij K.J.H., Vink J.M., Abdellaoui A., Gillespie N.A., Derks E.M., Treur J.L. (2022). The genetic aetiology of cannabis use: From twin models to genome-wide association studies and beyond. Transl. Psychiatry.

[137] Verweij K.J., Vinkhuyzen A.A., Benyamin B., Lynskey M.T., Quaye L., Agrawal A., Gordon S.D., Montgomery G.W., Madden P.A., Heath A.C. (2013). The genetic aetiology of cannabis use initiation: A meta-analysis of genome-wide association studies and a SNP-based heritability estimation. Addict. Biol..

[138] Gillespie N.A., Neale M.C., Kendler K.S. (2009). Pathways to cannabis abuse: A multi-stage model from cannabis availability, cannabis initiation and progression to abuse. Addiction.

[139] Berrettini W. (2017). A brief review of the genetics and pharmacogenetics of opioid use disorders. Dialogues Clin. Neurosci..

[140] Mistry C.J., Bawor M., Desai D., Marsh D.C., Samaan Z. (2014). Genetics of Opioid Dependence: A Review of the Genetic Contribution to Opioid Dependence. Curr. Psychiatry Rev..

[141] Wang L., Kranzler H.R., Gelernter J., Zhou H. (2025). Investigating the Contribution of Coding Variants in Alcohol Use Disorder Using Whole-Exome Sequencing Across Ancestries. Biol. Psychiatry.

[142] Song W., Kossowsky J., Torous J., Chen C.Y., Huang H., Mukamal K.J., Berde C.B., Bates D.W., Wright A. (2020). Genome-wide association analysis of opioid use disorder: A novel approach using clinical data. Drug Alcohol Depend..

[143] Legaki E., Tsaklakidou D., Hatzimanolis A., Segredou E., Petalotis M., Moularogiorgou G., Mouchtouri V., Lykouras L., Stefanis N.C., Gazouli M. (2022). Association of Alcohol Use Disorder Risk with ADH1B, DRD2, FAAH, SLC39A8, GCKR, and PDYN Genetic Polymorphisms. In Vivo.

[144] Davey Smith G., Hemani G. (2014). Mendelian randomization: Genetic anchors for causal inference in epidemiological studies. Hum. Mol. Genet..

[145] Richmond R.C., Davey Smith G. (2022). Mendelian randomization: Concepts and scope. Cold Spring Harb. Perspect. Med..

[146] Davies N.M., Holmes M.V., Davey Smith G. (2018). Reading Mendelian randomization studies: A guide, glossary, and checklist for clinicians. BMJ.

[147] Brumpton B., Sanderson E., Heilbron K., Hartwig F.P., Harrison S., Vie G.Å., Cho Y., Howe L.D., Hughes A., Boomsma D.I. (2020). Avoiding dynastic, assortative mating, and population stratification biases in Mendelian randomization through within-family analyses. Nat. Commun..

[148] Howe L.J., Nivard M.G., Morris T.T., Hansen A.F., Rasheed H., Cho Y., Chittoor G., Ahlskog R., Lind P.A., Palviainen T. (2022). Within-sibship genome-wide association analyses decrease bias in estimates of direct genetic effects. Nat. Genet..

[149] Hwang L.D., Davies N.M., Warrington N.M., Evans D.M. (2021). Integrating family-based and Mendelian randomization designs. Cold Spring Harb. Perspect. Med..

[150] Rosoff D.B., Clarke T.K., Adams M.J., McIntosh A.M., Davey Smith G., Jung J., Lohoff F.W. (2021). Educational attainment impacts drinking behaviors and risk for alcohol dependence: Results from a two-sample Mendelian randomization study with ~780,000 participants. Mol. Psychiatry.

[151] Burton S.M.I., Sallis H.M., Hatoum A.S., Munafò M.R., Reed Z.E. (2022). Is there a causal relationship between executive function and liability to mental health and substance use? A Mendelian randomization approach. R. Soc. Open Sci..

[152] Andrews S.J., Goate A., Anstey K.J. (2020). Association between alcohol consumption and Alzheimer’s disease: A Mendelian randomization study. Alzheimer’s Dement..

[153] Wootton R.E., Greenstone H.S.R., Abdellaoui A., Denys D., Verweij K.J.H., Munafò M.R., Treur J.L. (2021). Bidirectional effects between loneliness, smoking and alcohol use: Evidence from a Mendelian randomization study. Addiction.

[154] Lim K.X., Rijsdijk F., Hagenaars S.P., Socrates A., Choi S.W., Coleman J.R.I., Glanville K.P., Lewis C.M., Pingault J.B. (2020). Studying individual risk factors for self-harm in the UK Biobank: A polygenic scoring and Mendelian randomisation study. PLoS Med..

[155] Colbert S.M.C., Hatoum A.S., Shabalin A., Li Q.S., Coon H., Nelson E.C., Agrawal A., Docherty A.R., Johnson E.C. (2021). Exploring the genetic overlap of suicide-related behaviors and substance use disorders. Am. J. Med. Genet. B Neuropsychiatr. Genet..

[156] Treur J.L., Munafò M.R., Logtenberg E., Wiers R.W., Verweij K.J.H. (2021). Using Mendelian randomization analysis to better understand the relationship between mental health and substance use: A systematic review. Psychol. Med..

[157] Bountress K.E., Wendt F., Bustamante D., Agrawal A., Webb B., Gillespie N., Edenberg H., Sheerin C., Johnson E., Psychiatric Genomics Consortium Posttraumatic Stress Disorder Working Group (2021). Potential causal effect of posttraumatic stress disorder on alcohol use disorder and alcohol consumption in individuals of European descent: A Mendelian Randomization Study. Alcohol Clin. Exp. Res..

[158] Polimanti R., Peterson R.E., Ong J.S., MacGregor S., Edwards A.C., Clarke T.K., Frank J., Gerring Z., Gillespie N.A., Lind P.A. (2019). Evidence of causal effect of major depression on alcohol dependence: Findings from the psychiatric genomics consortium. Psychol. Med..

[159] Chen D., Wang X., Huang T., Jia J. (2023). Genetic support of a causal relationship between cannabis use and educational attainment: A two-sample Mendelian randomization study of European ancestry. Addiction.

[160] Johnson E.C., Hatoum A.S., Deak J.D., Polimanti R., Murray R.M., Edenberg H.J., Gelernter J., Di Forti M., Agrawal A. (2021). The relationship between cannabis and schizophrenia: A genetically informed perspective. Addiction.

[161] Gillespie N.A., Kendler K.S. (2021). Use of genetically informed methods to clarify the nature of the association between cannabis use and risk for schizophrenia. JAMA Psychiatry.

[162] Li H., Zhang X., Zhang X., Wang Z., Feng S., Zhang G. (2023). Can intelligence affect alcohol-, smoking-, and physical activity-related behaviors? A Mendelian randomization study. J. Intell..

[163] Vink J.M., Treur J.L., Pasman J.A., Schellekens A. (2021). Investigating genetic correlation and causality between nicotine dependence and ADHD in a broader psychiatric context. Am. J. Med. Genet. B Neuropsychiatr. Genet..

[164] Koob G.F., Volkow N.D. (2016). Neurobiology of addiction: A neurocircuitry analysis. Lancet Psychiatry.

[165] Alhammad M., Aljedani R., Alsaleh M., Atyia N., Alsmakh M., Alfaraj A., Alkhunaizi A., Alwabari J., Alzaidi M. (2022). Family, Individual, and Other Risk Factors Contributing to Risk of Substance Abuse in Young Adults: A Narrative Review. Cureus.

[166] Volkow N.D., Michaelides M., Baler R. (2019). The neuroscience of drug reward and addiction. Physiol. Rev..

[167] Ross S., Peselow E. (2012). Co-occurring psychotic and addictive disorders: Neurobiology and diagnosis. Clin. Neuropharmacol..

[168] Few L.R., Grant J.D., Trull T.J., Statham D.J., Martin N.G., Lynskey M.T., Agrawal A. (2014). Genetic variation in personality traits explains genetic overlap between borderline personality features and substance use disorders. Addiction.

[169] Lüscher C., Robbins T.W., Everitt B.J. (2020). The transition to compulsion in addiction. Nat. Rev. Neurosci..

[170] Davis C., Loxton N.J. (2013). Addictive behaviors and addiction-prone personality traits: Associations with a dopamine multilocus genetic profile. Addict. Behav..

[171] Bevilacqua L., Goldman D. (2013). Genetics of impulsive behaviour. Philos. Trans. R. Soc. Lond. B Biol. Sci..

[172] Kreek M.J., Nielsen D.A., Butelman E.R., LaForge K.S. (2005). Genetic influences on impulsivity, risk taking, stress responsivity and vulnerability to drug abuse and addiction. Nat. Neurosci..

[173] Teh L.K., Izuddin A.F., Fazleen M.H.H., Zakaria Z.A., Salleh M.Z. (2012). Tridimensional personalities and polymorphism of dopamine D2 receptor among heroin addicts. Biol. Res. Nurs..

[174] Sun X., Luquet S., Small D.M. (2017). DRD2: Bridging the Genome and Ingestive Behavior. Trends Cogn. Sci..

[175] Dalley J.W., Roiser J.P. (2012). Dopamine, serotonin and impulsivity. Neuroscience.

[176] Rodríguez-Cintas L., Daigre C., Grau-López L., Barral C., Pérez-Pazos J., Voltes N., Braquehais M.D., Casas M., Roncero C. (2016). Impulsivity and addiction severity in cocaine and opioid dependent patients. Addict. Behav..

[177] Dash G.F., Martin N.G., Slutske W.S. (2023). Big Five personality traits and illicit drug use: Specificity in trait-drug associations. Psychol. Addict. Behav..

[178] Maciocha F., Suchanecka A., Chmielowiec K., Chmielowiec J., Ciechanowicz A., Boroń A. (2024). Correlations of the *CNR1* Gene with Personality Traits in Women with Alcohol Use Disorder. Int. J. Mol. Sci..

[179] Recław R., Chmielowiec K., Suchanecka A., Boroń A., Chmielowiec J., Strońska-Pluta A., Kowalski M.T., Masiak J., Trybek G., Grzywacz A. (2024). The Influence of Genetic Polymorphic Variability of the Catechol-O-methyltransferase Gene in a Group of Patients with a Diagnosis of Behavioural Addiction, including Personality Traits. Genes.

[180] Aghaii S.H., Kamaly A., Esfahani M. (2012). Meta-Analysis of Individual and Environmental Factors that Influence People’s Addiction Tendencies. Int. J. High Risk Behav. Addict..

[181] Hines L.A., Morley K.I., Mackie C., Lynskey M. (2015). Genetic and Environmental Interplay in Adolescent Substance Use Disorders. Curr. Addict. Rep..

[182] Borrego-Ruiz A., Borrego J.J. (2024). Epigenetic Mechanisms in Aging: Extrinsic Factors and Gut Microbiome. Genes.

[183] Dai W., Qiao X., Fang Y., Guo R., Bai P., Liu S., Li T., Jiang Y., Wei S., Na Z. (2024). Epigenetics-targeted drugs: Current paradigms and future challenges. Signal Transduct. Target. Ther..

[184] Moore L.D., Le T., Fan G. (2013). DNA methylation and its basic function. Neuropsychopharmacology.

[185] Stillman B. (2018). Histone modifications: Insights into their influence on gene expression. Cell.

[186] Bannister A.J., Kouzarides T. (2011). Regulation of chromatin by histone modifications. Cell Res..

[187] Ngo A.L., Ahmad C.M., Gharavi Alkhansari N., Nguyen L., Zhang H. (2025). Epigenetic Insights into Substance Use Disorder and Associated Psychiatric Conditions. Complex Psychiatry.

[188] Ratti M., Lampis A., Ghidini M., Salati M., Mirchev M.B., Valeri N., Hahne J.C. (2020). MicroRNAs (miRNAs) and long non-coding RNAs (lncRNAs) as new tools for cancer therapy: First steps from bench to bedside. Target. Oncol..

[189] Jiang S., Postovit L., Cattaneo A., Binder E.B., Aitchison K.J. (2019). Epigenetic Modifications in Stress Response Genes Associated with Childhood Trauma. Front. Psychiatry.

[190] Markunas C.A., Semick S.A., Quach B.C., Tao R., Deep-Soboslay A., Carnes M.U., Bierut L.J., Hyde T.M., Kleinman J.E., Johnson E.O. (2021). Genome-wide DNA methylation differences in nucleus accumbens of smokers vs. nonsmokers. Neuropsychopharmacology.

[191] Lohoff F.W., Roy A., Jung J., Longley M., Rosoff D.B., Luo A., O’Connell E., Sorcher J.L., Sun H., Schwandt M. (2021). Epigenome-wide association study and multi-tissue replication of individuals with alcohol use disorder: Evidence for abnormal glucocorticoid signaling pathway gene regulation. Mol. Psychiatry.

[192] Lohoff F.W., Clarke T.K., Kaminsky Z.A., Walker R.M., Bermingham M.L., Jung J., Morris S.W., Rosoff D., Campbell A., Barbu M. (2022). Epigenome-wide association study of alcohol consumption in N = 8161 individuals and relevance to alcohol use disorder pathophysiology: Identification of the cystine/glutamate transporter SLC7A11 as a top target. Mol. Psychiatry.

[193] Fang F., Quach B., Lawrence K.G., van Dongen J., Marks J.A., Lundgren S., Lin M., Odintsova V.V., Costeira R., Xu Z. (2024). Trans-ancestry epigenome-wide association meta-analysis of DNA methylation with lifetime cannabis use. Mol. Psychiatry.

[194] Pepke M.L., Hansen S.B., Limborg M.T. (2024). Unraveling host regulation of gut microbiota through the epigenome-microbiome axis. Trends Microbiol..

[195] Hullar M.A., Fu B.C. (2014). Diet, the gut microbiome, and epigenetics. Cancer J..

[196] Strandwitz P. (2018). Neurotransmitter modulation by the gut microbiota. Brain Res..

[197] Walker D.M., Cates H.M., Loh Y.E., Purushothaman I., Ramakrishnan A., Cahill K.M., Lardner C.K., Godino A., Kronman H.G., Rabkin J. (2018). Cocaine Self-Administration Alters Transcriptome-wide Responses in the Brain’s Reward Circuitry. Biol. Psychiatry.

[198] Kolli U., Roy S. (2023). The role of the gut microbiome and microbial metabolism in mediating opioid-induced changes in the epigenome. Front. Microbiol..

[199] Barkus A., Baltrūnienė V., Baušienė J., Baltrūnas T., Barkienė L., Kazlauskaitė P., Baušys A. (2024). The Gut-Brain Axis in Opioid Use Disorder: Exploring the Bidirectional Influence of Opioids and the Gut Microbiome-A Comprehensive Review. Life.

[200] Cruz-Lebron A., Johnson R., Mazahery C., Troyer Z., Joussef-Pina S., Quinones-Mateu M.E., Strauch C.M., Hazen S.L., Levine A.D. (2021). Chronic opioid use modulates human enteric microbiota and intestinal barrier integrity. Gut Microbes.

[201] Du Y., Li L., Gong C., Li T., Xia Y. (2022). The diversity of the intestinal microbiota in patients with alcohol use disorder and its relationship to alcohol consumption and cognition. Front. Psychiatry.

[202] Ling Z., Zhu M., Yan X., Cheng Y., Shao L., Liu X., Jiang R., Wu S. (2021). Structural and functional dysbiosis of fecal microbiota in Chinese patients with Alzheimer’s disease. Front. Cell Dev. Biol..

[203] Bjørkhaug S.T., Aanes H., Neupane S.P., Bramness J.G., Malvik S., Henriksen C., Skar V., Medhus A.W., Valeur J. (2019). Characterization of gut microbiota composition and functions in patients with chronic alcohol overconsumption. Gut Microbes.

[204] Litwinowicz K., Choroszy M., Waszczuk E. (2020). Changes in the composition of the human intestinal microbiome in alcohol use disorder: A systematic review. Am. J. Drug Alcohol Abus..

[205] Vijay A., Kouraki A., Gohir S., Turnbull J., Kelly A., Chapman V., Barrett D.A., Bulsiewicz W.J., Valdes A.M. (2021). The anti-inflammatory effect of bacterial short chain fatty acids is partially mediated by endocannabinoids. Gut Microbes.

[206] Borrego-Ruiz A., Borrego J.J. (2025). A Holistic Review of Cannabis and Its Potential Risks and Benefits in Mental Health. Psychiatry Int..

[207] Biedermann L., Zeitz J., Mwinyi J., Sutter-Minder E., Rehman A., Ott S.J., Steurer-Stey C., Frei A., Frei P., Scharl M. (2013). Smoking cessation induces profound changes in the composition of the intestinal microbiota in humans. PLoS ONE.

[208] Shanahan E.R., Shah A., Koloski N., Walker M.M., Talley N.J., Morrison M., Holtmann G.J. (2018). Influence of cigarette smoking on the human duodenal mucosa-associated microbiota. Microbiome.

[209] Stewart C.J., Auchtung T.A., Ajami N.J., Velasquez K., Smith D.P., De La Garza R., Salas R., Petrosino J.F. (2018). Effects of tobacco smoke and electronic cigarette vapor exposure on the oral and gut microbiota in humans: A pilot study. PeerJ.

[210] Savin Z., Kivity S., Yonath H., Yehuda S. (2018). Smoking and the intestinal microbiome. Arch. Microbiol..

[211] Rueda-Ruzafa L., Cruz F., Cardona D., Hone A.J., Molina-Torres G., Sanchez-Labraca N., Roman P. (2020). Opioid system influences gut-brain axis: Dysbiosis and related alterations. Pharmacol. Res..

[212] Taboun Z.S., Sadeghi J. (2023). The bidirectional relationship between opioids and the gut microbiome: Implications for opioid tolerance and clinical interventions. Int. Immunopharmacol..

[213] Vincent C., Miller M.A., Edens T., Mehrotra S., Dewar K., Manges A.R. (2016). Bloom and bust: Intestinal microbiota dynamics in response to hospital exposures and *Clostridium difficile* colonization or infection. Microbiome.

[214] Acharya C., Betrapally N.S., Gillevet P.M., Sterling R.K., Akbarali H., Ganapathy D., Fagan A., Sikaroodi M., Bajaj J.S. (2017). Chronic opioid use is associated with altered gut microbiota and predicts readmissions in patients with cirrhosis. Aliment. Pharmacol. Ther..

[215] Xu Y., Xie Z., Wang H., Shen Z., Guo Y., Gao Y., Chen X., Wu Q., Li X., Wang K. (2017). Bacterial diversity of intestinal microbiota in patients with substance use disorders revealed by 16S rRNA gene deep sequencing. Sci. Rep..

[216] Barengolts E., Green S.J., Eisenberg Y., Akbar A., Reddivari B., Layden B.T., Dugas L., Chlipala G. (2018). Gut microbiota varies by opioid use, circulating leptin and oxytocin in African American men with diabetes and high burden of chronic disease. PLoS ONE.

[217] Herlihy B., Roy S. (2022). Gut-microbiome implications in opioid use disorder and related behaviors. Adv. Drug Alcohol Res..

[218] Wang F., Meng J., Zhang L., Johnson T., Chen C., Roy S. (2018). Morphine induces changes in the gut microbiome and metabolome in a morphine dependence model. Sci. Rep..

[219] Kilford E.J., Garrett E., Blakemore S.J. (2016). The development of social cognition in adolescence: An integrated perspective. Neurosci. Biobehav. Rev..

[220] Lamblin M., Murawski C., Whittle S., Fornito A. (2017). Social connectedness, mental health and the adolescent brain. Neurosci. Biobehav. Rev..

[221] Borrego-Ruiz A., Borrego J.J. (2024). Neurodevelopmental Disorders Associated with Gut Microbiome Dysbiosis in Children. Children.

[222] Zhang S., Wu S., Wu Q., Durkin D.W., Marsiglia F.F. (2021). Adolescent drug use initiation and transition into other drugs: A retrospective longitudinal examination across race/ethnicity. Addict. Behav..

[223] García-Cabrerizo R., Keller B., García-Fuster M.J. (2015). Hippocampal cell fate regulation by chronic cocaine during periods of adolescent vulnerability: Consequences of cocaine exposure during adolescence on behavioral despair in adulthood. Neuroscience.

[224] Spear L.P. (2016). Consequences of adolescent use of alcohol and other drugs: Studies using rodent models. Neurosci. Biobehav. Rev..

[225] Bis-Humbert C., García-Cabrerizo R., García-Fuster M.J. (2021). Increased negative affect when combining early-life maternal deprivation with adolescent, but not adult, cocaine exposure in male rats: Regulation of hippocampal FADD. Psychopharmacology.

[226] Baracz S.J., Everett N.A., Cornish J.L. (2020). The impact of early life stress on the central oxytocin system and susceptibility for drug addiction: Applicability of oxytocin as a pharmacotherapy. Neurosci. Biobehav. Rev..

[227] Eisenberger N.I., Moieni M., Inagaki T.K., Muscatell K.A., Irwin M.R. (2017). In Sickness and in Health: The Co-Regulation of Inflammation and Social Behavior. Neuropsychopharmacology.

[228] Buisman-Pijlman F.T.A., Sumracki N.M., Gordon J.J., Hull P.R., Carter C.S., Tops M. (2014). Individual differences underlying susceptibility to addiction: Role for the endogenous oxytocin system. Pharmacol. Biochem. Behav..

[229] Borrego-Ruiz A., Borrego J.J. (2025). Early-life gut microbiome development and its potential long-term impact on health outcomes. Microbiome Res. Rep..

[230] Borrego-Ruiz A., Borrego J.J. (2025). Early Life Stress and Gut Microbiome Dysbiosis: A Narrative Review. Stresses.

[231] Méndez Leal A.S., Silvers J.A. (2021). Neurobiological markers of resilience to early-life adversity during adolescence. Biol. Psychiatry. Cogn. Neurosci. Neuroimaging.

[232] Wolitzky-Taylor K., Sewart A., Vrshek-Schallhorn S., Zinbarg R., Mineka S., Hammen C., Bobova L., Adam E.K., Craske M.G. (2017). The effects of childhood and adolescent adversity on substance use disorders and poor health in early adulthood. J. Youth Adolesc..

[233] Lee J., Choi M., Holland M.M., Radey M., Tripodi S.J. (2022). Childhood Bullying Victimization, Substance Use and Criminal Activity among Adolescents: A Multilevel Growth Model Study. Int. J. Environ. Res. Public Health.

[234] Borrego-Ruiz A., Fernández S. (2024). Humiliation and its relationship with bullying victimization: A narrative review. Psychol. Soc. Educ..

[235] Burke A.R., DeBold J.F., Miczek K.A. (2016). CRF type 1 receptor antagonism in ventral tegmental area of adolescent rats during social defeat: Prevention of escalated cocaine self-administration in adulthood and behavioral adaptations during adolescence. Psychopharmacology.

[236] Montagud-Romero S., Aguilar M.A., Maldonado C., Manzanedo C., Miñarro J., Rodríguez-Arias M. (2015). Acute social defeat stress increases the conditioned rewarding effects of cocaine in adult but not in adolescent mice. Pharmacol. Biochem. Behav..

[237] Rodriguez-Arias M., Navarrete F., Blanco-Gandia M.C., Arenas M.C., Bartoll-Andrés A., Aguilar M.A., Rubio G., Miñarro J., Manzanares J. (2016). Social defeat in adolescent mice increases vulnerability to alcohol consumption. Addict. Biol..

[238] Ringwald W.R., Nielsen S.R., Mostajabi J., Vize C.E., van den Berg T., Manuck S.B., Marsland A.L., Wright A.G.C. (2024). Characterizing Stress Processes by Linking Big Five Personality States, Traits, and Day-to-Day Stressors. J. Res. Pers..

[239] Ballestín R., Alegre-Zurano L., Ferrer-Pérez C., Cantacorps L., Miñarro J., Valverde O., Rodríguez-Arias M. (2021). Neuroinflammatory and behavioral susceptibility profile of mice exposed to social stress towards cocaine effects. Prog. Neuro-Psychopharmacol. Biol. Psychiatry.

[240] Felger J., Treadway M. (2017). Inflammation Effects on Motivation and Motor Activity: Role of Dopamine. Neuropsychopharmacology.

[241] Martins D., Harrison N.A. (2025). Cytokines as neuromodulators: Insights from experimental studies in humans and non-human primates. Biol. Psychiatry.

[242] El Rawas R., Amaral I.M., Hofer A. (2020). Social interaction reward: A resilience approach to overcome vulnerability to drugs of abuse. Eur. Neuropsychopharmacol..

[243] Wemm S.E., Sinha R. (2019). Drug-induced stress responses and addiction risk and relapse. Neurobiol. Stress.

[244] Fosnocht A.Q., Briand L.A. (2016). Substance use modulates stress reactivity: Behavioral and physiological outcomes. Physiol. Behav..

[245] Parrott A.C., Sands H.R., Jones L., Clow A., Evans P., Downey L.A., Stalder T. (2014). Increased cortisol levels in hair of recent Ecstasy/MDMA users. Eur. Neuropsychopharmacol..

[246] Heilig M., Epstein D.H., Nader M.A., Shaham Y. (2016). Time to connect: Bringing social context into addiction neuroscience. Nat. Rev. Neurosci..

[247] Kelly J.F., Dow S.J., Westerhoff C. (2010). Does our choice of substance-related terms influence perceptions of treatment need? An empirical investigation with two commonly used terms. J. Drug Issues.

[248] Earnshaw V.A. (2020). Stigma and substance use disorders: A clinical, research, and advocacy agenda. Am. Psychol..

[249] Christie N.C. (2021). The role of social isolation in opioid addiction. Soc. Cogn. Affect. Neurosci..

[250] Borrego-Ruiz A. (2024). A current overview on adolescent alcohol misuse and its potential negative impacts. Alcohol. Drug Addict./Alkohol. Narkom..

[251] García-Cabrerizo R., Barros-Santos T., Campos D., Cryan J.F. (2023). The gut microbiota alone and in combination with a social stimulus regulates cocaine reward in the mouse. Brain Behav. Immun..

[252] Borrego-Ruiz A., Borrego J.J. (2024). An updated overview on the relationship between human gut microbiome dysbiosis and psychiatric and psychological disorders. Prog. Neuropsychopharmacol. Biol. Psychiatry.

[253] Cryan J.F., Dinan T.G. (2012). Mind-altering microorganisms: The impact of the gut microbiota on brain and behaviour. Nat. Rev. Neurosci..

[254] Collins S.M., Kassam Z., Bercik P. (2013). The adoptive transfer of behavioral phenotype via the intestinal microbiota: Experimental evidence and clinical implications. Curr. Opin. Microbiol..

[255] Bercik P. (2011). The intestinal microbiota affect central levels of brain-derived neurotropic factor and behavior in mice. Gastroenterology.

[256] Kelly J.R. (2016). Transferring the blues: Depression-associated gut microbiota induces neurobehavioural changes in the rat. J. Psychiatr. Res..

[257] Kim H.N., Yun Y., Ryu S., Chang Y., Kwon M.J., Cho J., Shin H., Kim H.L. (2018). Correlation between gut microbiota and personality in adults: A cross-sectional study. Brain Behav. Immun..

[258] Johnson K.V. (2020). Gut microbiome composition and diversity are related to human personality traits. Hum. Microb. J..

[259] Park E., Yun K.E., Kim M.H., Kim J., Chang Y., Ryu S., Kim H.L., Kim H.N., Jung S.C. (2021). Correlation between Gut Microbiota and Six Facets of Neuroticism in Korean Adults. J. Pers. Med..

[260] Borrego-Ruiz A., Borrego J.J. (2025). The Role of the Gut Microbiome on Borderline Personality Disorder. Behav. Psychol..

[261] Aatsinki A.K., Lahti L., Uusitupa H.M., Munukka E., Keskitalo A., Nolvi S., O’Mahony S., Pietilä S., Elo L.L., Eerola E. (2019). Gut microbiota composition is associated with temperament traits in infants. Brain Behav. Immun..

[262] Christian L.M., Galley J.D., Hade E.M., Schoppe-Sullivan S., Kamp Dush C., Bailey M.T. (2015). Gut microbiome composition is associated with temperament during early childhood. Brain Behav. Immun..

[263] Renson A., Kasselman L.J., Dowd J.B., Waldron L., Jones H.E., Herd P. (2020). Gut bacterial taxonomic abundances vary with cognition, personality, and mood in the Wisconsin Longitudinal Study. Brain Behav. Immun. Health.

[264] Aatsinki A.K., Kataja E.L., Munukka E., Lahti L., Keskitalo A., Korja R., Nolvi S., Häikiö T., Tarro S., Karlsson H. (2022). Infant fecal microbiota composition and attention to emotional faces. Emotion.

[265] Flannery J.E., Stagaman K., Burns A.R., Hickey R.J., Roos L.E., Giuliano R.J., Fisher P.A., Sharpton T.J. (2020). Gut Feelings Begin in Childhood: The Gut Metagenome Correlates with Early Environment, Caregiving, and Behavior. mBio.

[266] Callaghan B.L., Fields A., Gee D.G., Gabard-Durnam L., Caldera C., Humphreys K.L., Goff B., Flannery J., Telzer E.H., Shapiro M. (2020). Mind and gut: Associations between mood and gastrointestinal distress in children exposed to adversity. Dev. Psychopathol..

[267] Carlson A.L., Xia K., Azcarate-Peril M.A., Rosin S.P., Fine J.P., Mu W., Zopp J.B., Kimmel M.C., Styner M.A., Thompson A.L. (2021). Infant gut microbiome composition is associated with non-social fear behavior in a pilot study. Nat. Commun..

[268] Oyarzun J.P., Kuntz T.M., Stussi Y., Karaman O.T., Vranos S., Callaghan B.L., Huttenhower C., LeDoux J.E., Phelps E.A. (2022). Human threat learning is associated with gut microbiota composition. PNAS Nexus.

[269] Jia Y., Cheng S., Liu L., Cheng B., Liang C., Ye J., Chu X., Yao Y., Wen Y., Kafle O.P. (2023). Evaluating the Genetic Effects of Gut Microbiota on the Development of Neuroticism and General Happiness: A Polygenic Score Analysis and Interaction Study Using UK Biobank Data. Genes.

[270] Talarowska M.E., Kowalczyk M., Maes M., Carvalho A., Su K.P., Szemraj J., Gałecki P. (2020). Immune to happiness-inflammatory process indicators and depressive personality traits. Arch. Med. Sci..

[271] Delgadillo D.R., Pressman S.D., Christian L.M., Galley J.D., Bailey M.T. (2022). Associations Between Gut Microbes and Social Behavior in Healthy 2-Year-Old Children. Psychosom. Med..

[272] Sumich A., Heym N., Lenzoni S., Hunter K. (2022). Gut microbiome-brain axis and inflammation in temperament, personality and psychopathology. Curr. Opin. Behav. Sci..

[273] Wang Y., Chen X., Yu Y., Liu Y., Zhang Q., Bai J. (2020). Association between Gut Microbiota and Infant’s Temperament in the First Year of Life in a Chinese Birth Cohort. Microorganisms.

[274] GBD 2019 Diseases and Injuries Collaborators (2020). Global burden of 369 diseases and injuries in 204 countries and territories, 1990–2019: A systematic analysis for the Global Burden of Disease Study 2019. Lancet.

[275] Heather N. (2017). Is the concept of compulsion useful in the explanation or description of addictive behaviour and experience?. Addict. Behav. Rep..

[276] Hunt A., Merola G.P., Carpenter T., Jaeggi A.V. (2024). Evolutionary perspectives on substance and behavioural addictions: Distinct and shared pathways to understanding, prediction and prevention. Neurosci. Biobehav. Rev..

[277] Kedia Gupta S., Ambekar A., Dhawan A., Mehta M. (2017). Personality profile of alcohol and injecting opioid users: A comparative study from India. Asian J. Psychiatry.

[278] Wingo T., Nesil T., Choi J.S., Li M.D. (2016). Novelty Seeking and Drug Addiction in Humans and Animals: From Behavior to Molecules. J. Neuroimmune Pharmacol..

[279] Henden E., Melberg H.O., Røgeberg O.J. (2013). Addiction: Choice or compulsion?. Front. Psychiatry.

[280] Borrego-Ruiz A., Borrego J.J. (2025). Biological, Psychosocial, and Microbial Determinants of Childhood-Onset Obsessive–Compulsive Disorder: A Narrative Review. Children.

[281] Mackillop J. (2013). Integrating Behavioral Economics and Behavioral Genetics: Delayed Reward Discounting as an Endophenotype for Addictive Disorders. J. Exp. Anal. Behav..

[282] Winstanley C.A., Olausson P., Taylor J.R., Jentsch J.D. (2010). Insight Into the Relationship Between Impulsivity and Substance Abuse From Studies Using Animal Models. Alcohol. Clin. Exp. Res..

[283] Fineberg N.A., Potenza M.N., Chamberlain S.R., Berlin H.A., Menzies L., Bechara A., Sahakian B.J., Robbins T.W., Bullmore E.T., Hollander E. (2010). Probing Compulsive and Impulsive Behaviors, from Animal Models to Endophenotypes: A Narrative Review. Neuropsychopharmacology.

[284] Hamilton K.R., Mitchell M.R., Wing V.C., Balodis I.M., Bickel W.K., Fillmore M., Lane S.D., Lejuez C.W., Littlefield A.K., Luijten M. (2015). Choice Impulsivity: Definitions, Measurement Issues, and Clinical Implications. Pers. Disord..

[285] Andreassen C.S., Griffiths M.D., Gjertsen S.R., Krossbakken E., Kvam S., Pallesen S. (2013). The Relationships between Behavioral Addictions and the Five-Factor Model of Personality. J. Behav. Addict..

[286] Van Rooij A.J., Kuss D.J., Griffiths M.D., Shorter G.W., Schoenmakers T.M., Van De Mheen D. (2014). The (Co-) Occurrence of Problematic Video Gaming, Substance Use, and Psychosocial Problems in Adolescents. J. Behav. Addict..

[287] Badiani A., Belin D., Epstein D., Calu D., Shaham Y. (2011). Opiate versus Psychostimulant Addiction: The Differences Do Matter. Nat. Rev. Neurosci..

[288] Fernández-Serrano M.J., Pérez-García M., Verdejo-García A. (2011). What Are the Specific vs. Generalized Effects of Drugs of Abuse on Neuropsychological Performance?. Neurosci. Biobehav. Rev..

[289] Miller G.A., Rockstroh B. (2013). Endophenotypes in psychopathology research: Where do we stand?. Annu. Rev. Clin. Psychol..

[290] Liu C., Gershon E.S. (2024). Endophenotype 2.0: Updated definitions and criteria for endophenotypes of psychiatric disorders, incorporating new technologies and findings. Transl. Psychiatry.

[291] Chen Y.H., Hung T.W., Wang Y.S., Bae E.K., Wu K.J., Wang Y., Yu S.J. (2025). AAV-Mediated Expression of Methamphetamine Monoclonal Antibody Attenuates Methamphetamine Behaviour Sensitization in Mice. Addict. Biol..

[292] Li Y., Kong Q., Yue J., Gou X., Xu M., Wu X. (2019). Genome-edited skin epidermal stem cells protect mice from cocaine-seeking behaviour and cocaine overdose. Nat. Biomed. Eng..

[293] Hofford R.S., Meckel K.R., Wiser E.J., Wang W., Sens J.P., Kim M., Godino A., Lam T.T., Kiraly D.D. (2024). Microbiome depletion increases fentanyl self-administration and alters the striatal proteome through short-chain fatty acids. eNeuro.

[294] Volkow N.D., Blanco C. (2023). Substance use disorders: A comprehensive update of classification, epidemiology, neurobiology, clinical aspects, treatment and prevention. World Psychiatry.

[295] Gong Z., Xue Q., Luo Y., Yu B., Hua B., Liu Z. (2024). The interplay between the microbiota and opioid in the treatment of neuropathic pain. Front. Microbiol..

[296] Cooper S., Robison A.J., Mazei-Robison M.S. (2017). Reward circuitry in addiction. Neurotherapeutics.

[297] Zimmermann M., Zimmermann-Kogadeeva M., Wegmann R., Goodman A.L. (2019). Mapping human microbiome drug metabolism by gut bacteria and their genes. Nature.

[298] Lewin-Epstein O., Jaques Y., Feldman M.W., Kaufer D., Hadany L. (2023). Evolutionary modeling suggests that addictions may be driven by competition-induced microbiome dysbiosis. Commun. Biol..

[299] Gymrek M., Goren A. (2021). Missing Heritability May Be Hiding in Repeats. Science.

[300] Matthews L.J., Turkheimer E. (2022). Three Legs of the Missing Heritability Problem. Stud. Hist. Philos. Sci..

[301] Munafò M.R., Tilling K., Taylor A.E., Evans D.M., Davey Smith G. (2018). Collider scope: When selection bias can substantially influence observed associations. Int. J. Epidemiol..

[302] Xue A., Jiang L., Zhu Z., Wray N.R., Visscher P.M., Zeng J., Yang J. (2021). Genome-wide analyses of behavioural traits are subject to bias by misreports and longitudinal changes. Nat. Commun..

